# The novel family of transcendental Leal-functions with applications to science and engineering

**DOI:** 10.1016/j.heliyon.2020.e05418

**Published:** 2020-11-09

**Authors:** Hector Vazquez-Leal, Mario Alberto Sandoval-Hernandez, Uriel Filobello-Nino

**Affiliations:** a Facultad de Instrumentación Electrónica, Universidad Veracruzana, Cto. Gonzalo Aguirre Beltrán S/N, Xalapa, Veracruz, 91000, Mexico; b Consejo Veracruzano de Investigación Científica y Desarrollo Tecnológico (COVEICYDET), Av Rafael Murillo Vidal No. 1735, Cuauhtemoc, 91069, Xalapa, Veracruz, Mexico; c Universidad de Xalapa, Carretera Xalapa-Veracruz Km 2 No. 341, 91190, Xalapa, Veracruz, Mexico; d Instituto Tecnológico Superior de Poza Rica, Tecnológico Nacional de México, Luis Donaldo Colosio Murrieta S/N, Arroyo del Maíz, C.P. 93230 Poza Rica, Veracruz, Mexico

**Keywords:** Mathematics, Transcendental functions, Power Series Extender Method, Approximate methods

## Abstract

During the phenomena modelling process in the different areas of science and engineering is common to face nonlinear equations without exact solutions; thus, the need of employing numerical methods to obtain such solutions. Therefore, in order to provide new possibilities for the isolation of variables, we propose a novel family of transcendental functions with new algebraic properties including their integration and differentiation rules. Likewise, in order facilitate the numerical evaluation for every new family set of functions, a highly accurate series of approximations is proposed by employing analytical expressions in terms of known transcendental functions and polynomials combinations. By the use of known functions for the proposed approximations, makes possible the use of any programming language for their respective implementation. In this article, three interesting case studies are presented with applications on: coastal engineering, transmission lines span on electrical engineering, and the planar one-dimensional Bratu equation. Finally, based on the results from study cases, it can be concluded that Leal-functions will have relevant impact in all areas of physics and mathematics, by providing new tools to scientists and engineers for the proposal of new mathematical models and numerical/analytical analysis, design and implementation of new theories and technological innovations.

## 1 Introduction

The use of transcendental functions is widely employed in science and engineering because they allow to perform the modelling and simulation of physical phenomena. Importance of transcendental functions is given by the fact that they can be found, practically, in all mathematical analysis which has enhanced development of theoretical sciences and the creation of new technologies. Transcendental functions allow to perform the analysis of mechanical vibration in the field of physics [1], the analysis of alternating current in electrical circuits [1], [2], electro magnetics theory [3], quantum mechanics [4], optics [5], digital signal processing [6], electronic circuit oscillator generated waveform [7], the modelling of wave heat conduction [8], among others. Another application for transcendental functions, in the case of logarithms, is its use in Bode analysis [2], [9], [10] for the creation of phase and magnitude plots.

The development of mathematical tools to perform analysis such as Fourier [11], [12] and Laplace transform [1], [13], has been possible through transcendental functions. Furthermore, hyperbolic functions also play an important role in mathematical analysis for science and engineering; for instance, in civil engineering, they have applications in the study and design of catenary forms in chains and cables for suspended bridges [14], in electrical engineering for the design of free hanging electric power cables [15], [16], [17]; also, in naval and civil engineering, for the modelling of sea wave behaviour [18], among other applications.

Several historical records show that the use of trigonometry comes from ancient civilizations such as Hindu and Chinese [19]. For instance, in Babylonian civilization trigonometry was employed to perform agricultural measurements of land; also, it was used during pyramid construction in Egypt, showing how Egyptians had some knowledge of triangle numerical relations, that was called a kind of proto-trigonometry [20]. This knowledge was passed on to the Greeks and, around century II B.C., Hipparchus of Nicaea (190-120), known as the father of trigonometry, was the first to construct a trigonometric table chart.

Also, in the II century B.C., Claudius Ptolemy (85-165) continued the improvement of Hipparchus' works providing examples on how to utilize the tables on the solution of rectangle triangles. In India, the developing of trigonometric system was based on the sine function; one of the mayor contributions of this civilization was the development of the sexagesimal system. The first sine table ever constructed was found in Aryabhatiya, written by Aryabhata I (475-550); nevertheless, the word it sine appeared for the first time in the manuscripts of Gerard of Cremona (1114-1187) who translated texts from Greek to Latin, including the Almagest [20]. In Europe, after the end of the Middle Ages, in 1453 A.C. with the fall of Constantinople by the Ottoman Turk empire, Johannes Müller Von Königsberg (1436-1476), known as Regiomontanus, in [21] presented the methods to solve triangles [22], which started the renaissance of trigonometry. Besides, this work introduced the Law of Sines and included practical problems to determine triangle sides [22], [23]. By 1551 A.C., Georg Joachim Rheticus (1514-1574) presented the first publication of a trigonometric table that included the six elemental functions [24]. His work was the first to define the six trigonometric functions as ratios of a triangle sides; although, he used different names for the ratios. Thomas Fincke (1561-1556) proposed to abbreviate the trigonometric functions with a notation that included dots, which is the base for today's used notation [25]. In addition to that, Fincke introduces the terms *tangent*, *cotangent*, *secant*, and cosecant [26].

Along with the mathematical advancements, arithmetic symbols underwent changes. For instance, in 1557 Robert Recorde (1510-1558), mathematician and physician, introduced the equal sign (=) [27]. Nonetheless, equal sign will be used until 1618. Algebra also suffered modifications, François Viète (1540-1603) introduced in [28] the new algebra that consisted in the use of alphabet letters to represent variables, constant or indeterminate terms in any equation. In [29], Viète presents formulas related to *sines* and *cosines*, which gave place to the first trigonometry tables of our era since the Arab mathematicians in the tenth century. Also, in [29], algebra was applied to trigonometry. Viète discovered most of the elementary trigonometric identities and obtains the general formulas equivalent to the expressions of sin⁡(nx) and cos⁡(nx) in function of sin⁡(x) and cos⁡(x). Among important trigonometry identities, we have:

(1) sin⁡(A+B)+sin⁡(A−B)=2sin⁡(A)cos⁡(B),sin⁡(A−B)−sin⁡(A−B)=2sin⁡(B)cos⁡(A),cos⁡(A−B)+cos⁡(A+B)=2cos⁡(A)cos⁡(B).

Later on, trigonometric identities were employed to perform multiplication and division [30]. Works from Jost Börgi contributed to the development of new methods and trigonometric formulas employed in the Prostaphaeresis by the end of sixteenth century [31].

In the sixteenth century, arithmetical calculation became important due to the commercial expansion and perfection of navigation which made necessary to find less laborious algorithms, mainly for multiplication and division. This fact gave place to the logarithm discovery; although their beginning is from Archimedes' era [32]. In Edinburgh, by 1614, John Napier (1550-1617) published the first logarithm table [33]; in 1619, after Napier's death, the procedure for logarithm construction was published [34]. Henry Briggs in 1617 proposed the base-10 logarithm [35] and, in 1624, [36] presented an extended table that contained the logarithm for 30000 natural numbers with 14 significant digits. By 1620, Edmund Gunter (1581-1626) continued the works from Briggs and Napier, providing the tables [37], where the base-10 logarithm for *sine*, *cosine*, *tangent*, and *cotangent* trigonometric functions are calculated. Nevertheless, a few years before, Michael Stifel (1487-1567) in 1544 published a table of logarithms [38] that presented calculations with powers of an arbitrary rational exponent, and particularly, the multiplication rule given by

(2) $$a^{n}a^{m}=a^{n+m},$$

for rational numbers.

In 1748, L. Euler publishes [39], [40] which studies elementary functions and geometric curves, without recurring to integral and differential calculus. In there, he introduces the concept of function referring to the logarithmic, exponential, and trigonometric expressions. Besides, Euler presents the notation for *x* logarithm base *a* as it is known nowadays ($\log_{a}(x)$), and introduces the number *e* as the natural base [39]. Likewise, in [39], Euler presents the exponential and logarithm functions with their respective power series.

Euler classified addition, multiplication, subtraction, division, raising to a power, and *n*-th roots as algebraic operations; while classifying non algebraic operations as transcendentals [39], [41]. Thus, Euler labelled trigonometric, exponential, and logarithmic as transcendental functions [39], [41]; in this way, classified functions as algebraic and transcendental. It is important to note that nowadays, addition, multiplication, and division are considered as arithmetic operations [42] which are part of fundamental algebra.

Johann H. Lambert (1728-1777) was the first to publish a treatise of hyperbolic functions in 1761 [43]. The name of *hyperbolic function* comes from the comparison between a semi-circular surface area and a hyperbola section area [44]. In his work, Lambert did not introduce a notation for these functions; although, he did find the analogy between these functions and circular trigonometric functions [43], [44]. Lambert continued his work on hyperbolic functions and their similitude to circular trigonometric functions [44]. But it was until then that Lambert provided a notation for them, to be differentiated from the traditional trigonometric functions. Nevertheless, this notation was different as we use it nowadays by [44], [45] which includes an extensive list of multiple angles identities derived from:

(3) $$\sin\text{hyp}v=\frac{\exp(v)-\exp(-v)}{2},\cos\text{hyp}v=\frac{\exp(v)+\exp(-v)}{2},$$

where (3) is presented with the original notation proposed by Lambert in [44], [45].

The main reason for Lambert to work on hyperbolic functions was to simplify triangles sides and angles calculation. Besides that, Vincenzo Riccati (1707-1775) worked with hyperbolic functions, between 1757-1772, using a hyperbole to define functions that he called *sinus hyperbolico* and *cosinus hyperbolico* [46]. On his work, he also determined the representation of power series for these functions. In [47], Ricatti developed the theory of hyperbolic functions including some hyperbolic identities, their derivatives, and its relation to exponential function [45].

In 1758, Lambert solves the equation $x=q+x^{n}$ by giving a series development for *x* in powers of *q* [48]. Later, Euler modified it [49] to

(4) $$x^{\alpha }-b^{\beta }=(\alpha -\beta )vx^{\alpha +\beta }.$$

Works from Lambert and Eisenstein [50], among others, led to the development of Lambert function W(x), defined as the inverse of

(5) yexp⁡(y)=x,

its solution given by

(6) y=W(x).

The inverse of (5) was proposed by Pólya and Szegö in 1925 [51]. The convention to identify Lambert function with *W* was possible for the early works of Edward Maitland Wright and his contributions of delay differential equations (DDEs) [52], [53]. From 1949 to 1959 Wright had good results solving (5), which is the characteristic equation associated to DDE $x^{\prime }=Bx(t-r)$ [52], [53], [54]. Nevertheless, the use of Lambert *W* function started in 1973, when [55] presented a numerical algorithm to solve (5) coded in Fortran computer language. This algorithm allowed the calculation of real solutions when x>0. The importance of Lambert *W* function was not really appreciated until the 1990s when Corless and developers of the Maple mathematical software performed an exhaustive research and found that this function had practical applications [56], [57]. Table 1 outlines the most important historical events about transcendental functions.

**Table 1:** Main historical events of transcendental functions.

Year/Period	Historical Event	Author
B.C.	Trigonometry in ancient civilizations	Anonymous
	Mesopotamia, Egypt, China, India	
190-120	Constructed the first trigonometric table	Hipparchus of Nicaea
1464	Presents a triangle resolution procedure by Law of sines	J.M. Regiomontanus
1551	Six trigonometric functions are published for the first time	Georg Joachim R.
1540-1603	Introduces modern algebra notation	F. Vieta
1552-1632	His work contributed to Prosthaphaeresis calculus	Jost Bürgi
1614	Introduced the first logarithm tables	J. Naphier
1617	The first table of base-10 logarithm was compiled	H. Briggs
1620	Presented the first base-10 logarithmic tables for trigonometric functions	Edmund Gunter
1748	Defined the concept of function	L. Euler
1761	Introduced hyperbolic functions	J.H. Lambert
1765-1767	Developed properties of hyperbolic functions	V. Ricatti
1758	Solves Diophantine equation *x* = *q* + *x*^*n*^ (the direct antecedent of *W*(*x*))	J.H. Lambert
1925	The formal proposal for inverse of W(x)exp⁡(W(x)) is presented	Pólya Y Szegö
1990s	Algorithms to evaluate *W*(*x*) are implemented	Corless & Developers

Table 1 shows that existence of trigonometric functions dates back to ancient civilizations and played an important role in the determination of object dimensions. Furthermore, its evolution throughout the centuries is observed. Nevertheless, after Middle Ages, the improvement of these functions notoriously continued until 17th century, time when logarithms were born and in 1620 the logarithmic tables for traditional trigonometric functions were published in the works of Edmund Gunter. Besides, the works from Riccatti and Lambert were of high importance because they proposed hyperbolic functions and their properties, which initiates the modern use of transcendental functions with the notation that we know today. Later on, J.H. Lambert, in 1758, provided the principles for a new transcendental function (W(x)) that was almost unknown until 1990s, including its applications to science and engineering. This function was fully developed until 1925 by Pólya and Szegö [51] moreover, thanks to the algorithms implemented by Corless & developers [57], this function began to be widely employed by the end of 20th century.

In this article, we present a novel and interesting family of functions with new algebraic properties, which, from now on, we will denominate as Leal family of transcendental functions. In literature when it is encountered a nonlinear equation which is not possible to solve analytically for one of the variables, it is necessary the application of numerical algorithms like Newton-Raphson method, or regula-falsi method, among others [58]. Likewise, when solving a nonlinear differential equation, conventional techniques could not be employed, thus, numerical techniques such as Runge Kutta, among others [58], [59], [60], are employed. Nevertheless, the problem of using numerical methods is that in occasions they exhibit instabilities, oscillations, and divergences; besides of requiring a certain high computing power, among other consequences [58]. Another disadvantage in the use of numerical algorithms is due to the fact that these algorithms do not provide exact solutions that would allow performing other types of mathematical analysis or conclusions [10], [61], [62]. It is important to remark that the main reason of using numerical algorithms for the solution of nonlinear problems lies in the fact that known transcendental functions do not possess the necessary algebraic properties to obtain an exact solution. In consequence, a family of transcendental functions will be presented along with their respective differentiation and integration rules, with some of their properties and identities. A key aspect taken into account in this research will be the proposal of a series of analytic approximations that allows to evaluate the transcendental family of Leal-functions in terms of known transcendental functions and polynomial expressions. Such approximate functions have the advantage of being handy and easy computable, providing highly accurate results. These expressions will be obtained by using Power Series Extender Method (PSEM) [63], [64], [65], [66], which is simple to use and does not require either integral calculations or existence of perturbative parameters, among other characteristics.

This research article is organized as follows, Section 2 presents some characteristics of the known transcendental functions. Section 3 shows an introduction of PSEM. In Section 4, we introduce the family of Leal-functions. Section 5, indicates the process of approximation of Leal-functions. In Section 6, we present the procedure that is applied in all proposed functions in this work. Next, Section 7 presents three interesting applications. An algorithm to improve the accuracy of the PSEM approximations is presented in Section 8. Section 9 shows the computation time and discussion. Section 10 presents the concluding remarks and future work of this research.

## 2 Some basis of known transcendental and hyperbolic functions

**Figure 1:**
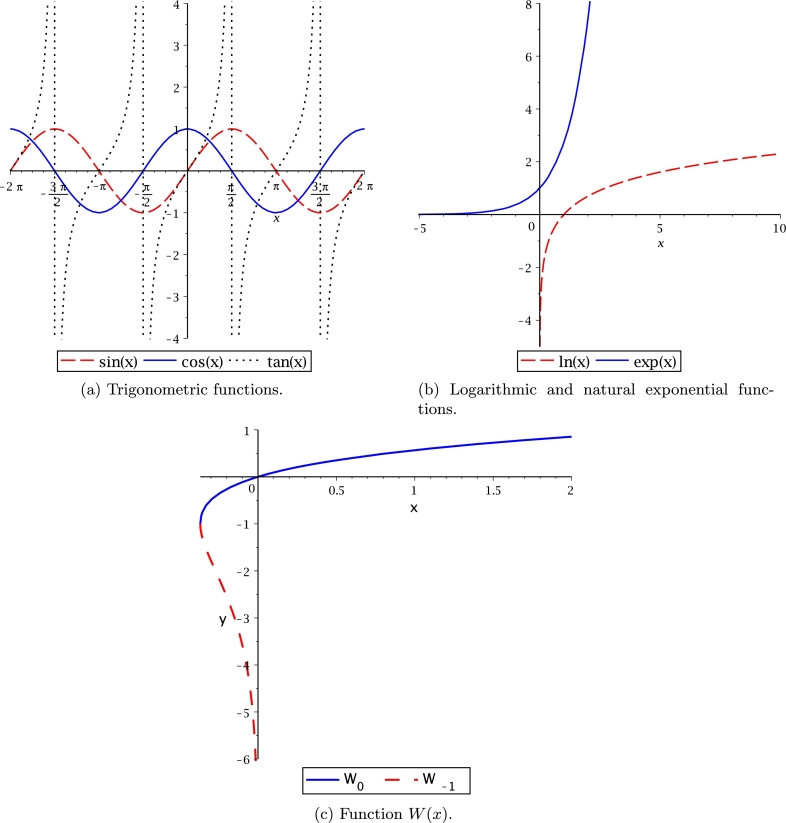
Transcendental functions.

Fig. 1(a) shows the group of transcendental functions for *sine*, *cosine* and *tangent*, where the period for each one is displayed; also, the continuity of *sine* and *cosine* functions, π/2 difference phase, the domain and co-domain are also depicted. *Tangent* function exhibits a periodic discontinuity every π/2. Fig. 1(b) illustrates natural logarithm and exponential functions, where it is observed the faster growing of the exponential function, while logarithm function is not defined at x=0. Also, these functions are inverse, as shown in the figure. Fig. 1(c) presents the function W(x) [56], [57] which is a multivalued function at −exp⁡(−1)≤x≤0. In addition, W(x) shows two real branches:

• $W_{-1}$ known as the lower branch that satisfies the condition W(x)<−1.
• $W_{0}$ known as the upper branch that satisfies the condition W(x)≥−1.

It is important to remark that both branches are jointed at (−exp⁡(−1),−1).

**Figure 2:**
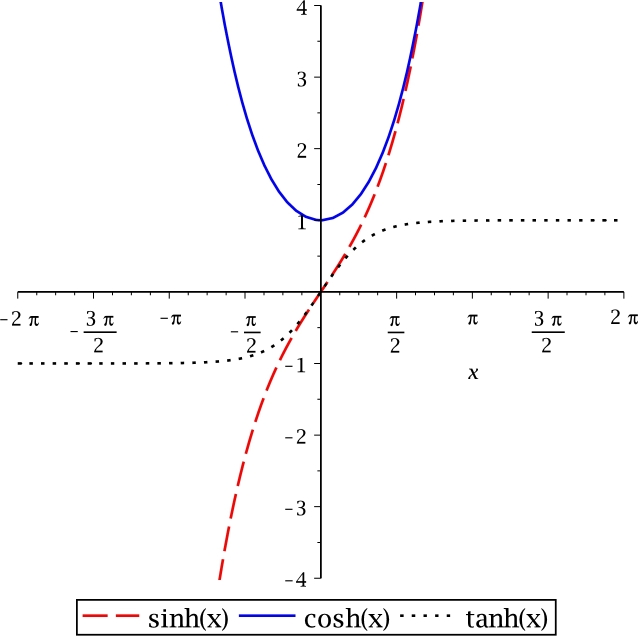
Hyperbolic functions.

The behaviour of hyperbolic functions (*sine*, *cosine* and *tangent*) is depicted in Fig. 2. The sinh⁡(x) and tanh⁡(x) cross at origin; nevertheless, as *x* tends to grow to the right, its range of numerical values tends to be the same. In contrast, tanh has an asymptotic behaviour at both branches within the limits −1 to +1.

Table 2 lists the domain, range, and period in the domain of real numbers for transcendental and hyperbolic functions; also, for their derivatives and integrals. In addition, periodicity, odd, and even symmetry for trigonometric functions are listed. Hyperbolic functions have odd and even symmetry but no periodicity. The W(x) function is the only transcendental multivalued function.

**Table 2:** Transcendental functions.

Function (x)	Domain	Range	Period	Parity	Derivative	Integral
sin(*x*)	−∞ < *x* < ∞	−1 ≤ *y* ≤ 1	2*π*	Odd	cos⁡(x)	−cos⁡(x)+C
cos(*x*)	−∞ < *x* < ∞	−1 ≤ *y* ≤ 1	2*π*	Even	−sin⁡(x)	sin⁡(x)+C
tan(*x*)	$-\frac{\pi }{2}<x<\frac{\pi }{2}$	−∞ < *y* < ∞	*π*	Odd	$\sec^{2}(x)$	ln⁡|sec(x)|+C
ln(*x*)	0 < *x* < ∞	−∞ < *y* < ∞	★	♠	$\frac{1}{x}$	x(ln⁡(x)−1)+C
exp(*x*)	−∞ < *y* < ∞	0 < *x* < ∞	★	♠	exp⁡(x)	exp⁡(x)+C
*W*_0_(*x*)	$-\frac{1}{e}\leq x\leq \infty$	−1 ≤ *y* < ∞	★	♠	$\frac{W(x)}{x(1+W(x))}$	$\frac{x(W^{2}(x)-W(x)+1)}{W(x)}+C$
*W*_−1_(*x*)	$-\frac{1}{e}\leq x<0$	−∞ < *y* ≤ −1	★	♠		
sinh(*x*)	−∞ < *x* < ∞	−∞ < *y* < ∞	★	Odd	cosh(*x*)	cosh⁡(x)+C
cosh(*x*)	−∞ < *x* < ∞	1 ≤ *y* < ∞	★	Even	sinh(*x*)	sinh⁡(x)+C
tanh(*x*)	−∞ < *x* < ∞	−1 < *y* < 1	★	Odd	sech^2^(*x*)	ln⁡|cosh⁡(x)|+C

★ It does not exhibit periodicity.

♠ It does not exhibit symmetry.

## 3 Basic concept of PSEM method

In broad sense, a nonlinear differential equation can be expressed as:

(7) L(u)+N(u)−f(x)=0,x∈Ω,

having as boundary condition

(8) $$B(u,\frac{\partial u}{\partial \eta })=0,\;x\in \Gamma ,$$

where *L* and *N* are a linear operator and a nonlinear operator, respectively; f(x) is a known analytic function; *B* ia a boundary operator; Γ is the boundary of domain Ω and $\frac{\partial u}{\partial \eta }$ denotes differentiation along the normal drawn outwards from Ω [67].

Next, the solution of (7) can be express as a Taylor expansion [68]

(9) $$u=\sum_{k=0}^{\infty }v_{k}{\left(x-x_{0}\right)}^{k},$$

where $v_{k}(k=0.1,2,\ldots )$ are coefficients of the power series and $x_{0}$ is the expansion point.

Now, in [63], [69], it is proposed that the solution for (7) can be written as a finite sum of functions as

(10) $$u=u_{0}+\sum_{i=0}^{n}f_{i}(x,u_{i}),$$

or

(11) $$u=\frac{u_{0}+\sum_{i=0}^{n}f_{i}(x,u_{i})}{1+\sum_{j=n+1}^{2n}f_{j}(x,u_{j})},$$

where $u_{i}$ are constants to be determined by PSEM; $f_{i}(x,u_{i})$ are arbitrary trial functions; *n* and 2*n* are the orders of approximations in (10) and (11), respectively. We will denominate (10) and (11) as a trial function (TF). Next, we calculate the Taylor expansion of (10) or (11), resulting in the power series:

(12) $$u=u_{0}+\sum_{i=0}^{n}P_{i,0}+\sum_{i=0}^{n}\sum_{k=1}^{\infty }P_{i,k}{\left(x-x_{0}\right)}^{k},$$

(13) $$u=u_{0}+\sum_{i=0}^{n}P_{i,0}+\sum_{i=0}^{2n}\sum_{k=1}^{\infty }P_{i,k}{\left(x-x_{0}\right)}^{k},$$

respectively, where Taylor coefficients $P_{k}$ are expressed in terms of parameters $u_{i}$. Finally, we equate/match the coefficients of power series either in (12) or (13) with the correspondent ones of (9) to obtain the values of $u_{i}$ and substitute them into (10) or (11), to obtain the PSEM approximation [63], [64], [65], [66].

It is possible to enrich PSEM by means of introducing cancellation points (C.P.). For every cancellation point, a new equation is included into PSEM process to complement the system of equations in order to obtain coefficients ($u_{i}$). The cancellation equation is constructed by evaluating the TF at some strategically selected C.P. and equating its result to the exact value (obtained numerically). It is important to notice that every introduced cancellation point would replace the corresponding superior order equation in the regular PSEM procedure.

The TF can be made-up combining different type of functions as long as Taylor series exist. Note that regrouping *x*-powers terms is valid for (12) and (13), because $f_{i}$ functions were chosen analytic in a well-defined domain for independent variable *x*; whereby, the correspondent Taylor series are convergent for such values of *x* [1], [70]. By restricting (7) values of the mentioned domain of convergence, the sum of Taylor series for $f_{i}$ is also convergent [1], [70]. In the same fashion, the quotient of two analytic functions series (see (11)) in $x_{0}$ is also analytic, whenever the denominator is different from zero at $x_{0}$. It is important to notice that PSEM convergence greatly depends on the proper selection of the trial function. Then, it is necessary that the proposed TF can potentially describe the qualitative behaviour of the nonlinear problem solution.

## 4 The novel family of Leal-functions

In this work, we propose the following novel family of Leal-functions:

**Definition** y(x)=Lsinh(x)**.** This symmetric Leal-function exhibits as its main property the capability to isolate the transcendental equation y(x)sinh⁡(y(x))=x. Furthermore, Lsinh(*x*) is defined by its series expansion at x=0

$$\text{Lsinh}(x)=\pm x^{1/2}\mp \frac{1}{12}x^{3/2}\pm \frac{29}{1440}x^{5/2}\mp \frac{263}{40320}x^{7/2}\pm \frac{23479}{9676800}x^{9/2}+\cdots ,\;x\geq 0,$$

for its both branches. It is important to note that we employed g(x)=x for all definitions of Leal-Functions.

**Definition** y(x)=Lcosh(x)**.** This Leal-function exhibits as its main property the capability to isolate the transcendental equation y(x)cosh⁡(y(x))=x. Furthermore, Lcosh(*x*) is defined by its series expansion at x=0

$$\text{Lcosh}(x)=x-\frac{1}{2}x^{3}+\frac{17x^{5}}{24}-\frac{961x^{7}}{720}+\frac{116129x^{9}}{40320}+\cdots ,\;x\in R.$$

**Definition** y(x)=Ltanh(x)**.** This symmetric Leal-function exhibits as its main property the capability to isolate the transcendental equation y(x)tanh⁡(y(x))=x. Furthermore, Ltanh(*x*) is defined by its series expansion at x=0

$$\text{Ltanh}(x)=\pm \sqrt{x}\pm \frac{1}{6}x^{3/2}\pm \frac{11x^{5/2}}{360}\pm \frac{17x^{7/2}}{5040}\mp \frac{281x^{9/2}}{604800}+\cdots ,\;x\geq 0,$$

for its both branches.

**Definition** y(x)=Lcsch(x)**.** This symmetric Leal-function exhibits as its main property the capability to isolate the transcendental equation y(x)csch(y(x))=x. Furthermore, Lcsch(*x*) is defined by its series expansion at x=1/2

$$\begin{array}{ccc} \text{Lcsch}(x)\; & =\; & \pm 2.17731900\mp 3.52890440(x-1/2)\pm 1.7907961{\left(x-1/2\right)}^{2}\\ \; & \; & \mp 4.0466882{\left(x-1/2\right)}^{3}\pm 3.8584093{\left(x-1/2\right)}^{4}\\ \; & \; & \mp 9.1280050{\left(x-1/2\right)}^{5}+\cdots ,\;0\leq x\leq 1, \end{array}$$

for its both branches.

**Definition** y(x)=Lsech(x)**.** This Leal-function exhibits as its main property the capability to isolate the transcendental equation y(x)sech(y(x))=x. Due to symmetries we will concentrate on the first quadrant where Lsech(*x*) can be split into two branches; the lower branch is defined by the following series expansion at x=0

$$\begin{array}{c} {\text{Lsech}}^{-}(x)=x+\frac{1}{2}x^{3}+\frac{13x^{5}}{24}+\frac{541x^{7}}{720}+\cdots ,\;0\leq x\leq \alpha ,\;0\leq y<\beta , \end{array}$$

where α=βsech(β) and *β* results from the solution of βtanh⁡(β)−1=0. It results that α=0.66274342 and β=1.19967864. For the case of the upper branch, the series expansion is defined at x=1/2 as

$$\begin{array}{ccc} {\text{Lsech}}^{+}(x)\; & =\; & 2.12679989-3.98579130(x-1/2)-0.38882963{\left(x-1/2\right)}^{2}\\ \; & \; & -12.80006750{\left(x-1/2\right)}^{3}-32.50338675{\left(x-1/2\right)}^{4}\\ \; & \; & -170.18895168{\left(x-1/2\right)}^{5}+\cdots ,\;0\leq x\leq \alpha ,\;\beta \leq y\leq \infty . \end{array}$$

**Definition** y(x)=Lcoth(x)**.** This symmetric Leal-function exhibits as its main property the capability to isolate the transcendental equation y(x)coth⁡(y(x))=x. Furthermore, Lcoth(*x*) is defined by its series expansion at x=3/2

$$\begin{array}{ccc} \text{Lcoth}(x)\; & =\; & \mp 1.28783945\pm 1.41749728(x-3/2)\mp 0.50786190{\left(x-3/2\right)}^{2}\\ \; & \; & \pm 0.58433233{\left(x-3/2\right)}^{3}\mp 0.74458185{\left(x-3/2\right)}^{4}\\ \; & \; & \pm 1.04948406{\left(x-3/2\right)}^{5}+\cdots ,\;x\geq 1, \end{array}$$

for its both branches.

**Definition** y(x)=Lln(x)**.** This asymmetric Leal-function exhibits as its main property the capability to isolate the transcendental equation y(x)ln(y(x)+1)=x. Lln(*x*) has two branches with the following series expansions at x=0

$$\begin{array}{c} {\text{Lln}}^{+}(x)=\sqrt{x}+x/4-\frac{x^{3/2}}{96}+\frac{59x^{5/2}}{92160}-\frac{x^{3}}{2880}+\cdots ,\;x\geq 0, \end{array}$$

and

$$\begin{array}{c} {\text{Lln}}^{-}(x)=-\sqrt{x}+x/4+\frac{x^{3/2}}{96}-\frac{59x^{5/2}}{92160}-\frac{x^{3}}{2880}+\cdots ,\;x\geq 0, \end{array}$$

for superior and inferior branches, respectively.

**Definition** y(x)=Ltan(x)**.** This symmetric Leal-function exhibits as its main property the capability to isolate the transcendental equation y(x)tan⁡(y(x))=x. Furthermore, Ltan(*x*) is defined by its series expansion at x=0

$$\text{Ltan}(x)=\pm \sqrt{x}\mp \frac{1}{6}x^{3/2}\pm \frac{11x^{5/2}}{360}\mp \frac{17x^{7/2}}{5040}\mp \frac{281x^{9/2}}{604800}+\cdots ,\;0\leq x<\infty ,-\pi /2<y<\pi /2,$$

for its both branches.

**Definition** $y(x)={\text{Lsinh}}_{2}(x)$**.** This Leal-function exhibits as its main property the capability to isolate the transcendental equation y(x)+sinh⁡(y(x))=x. Furthermore, ${\text{Lsinh}}_{2}(x)$ is defined by its series expansion at x=0

$${\text{Lsinh}}_{2}(x)=\frac{x}{2}-\frac{x^{3}}{96}+\frac{x^{5}}{1920}-\frac{43x^{7}}{1290240}+\cdots ,\;x\in R.$$

**Definition** $y(x)={\text{Lcosh}}_{2}(x)$**.** This Leal-function exhibits as its main property the capability to isolate the transcendental equation y(x)+cosh⁡(y(x))=x. Furthermore, we will define ${\text{Lcosh}}_{2}(x)$ by its series expansion at x=1 for the upper branch

$$\begin{array}{c} {\text{Lcosh}}_{2}(x)=(x-1)-\frac{1}{2}{\left(x-1\right)}^{2}+\frac{1}{2}{\left(x-1\right)}^{3}-\frac{2}{3}{\left(x-1\right)}^{4}+{\left(x-1\right)}^{5}+\cdots ,\\ \;x\geq 0. \end{array}$$

**Figure 3:**
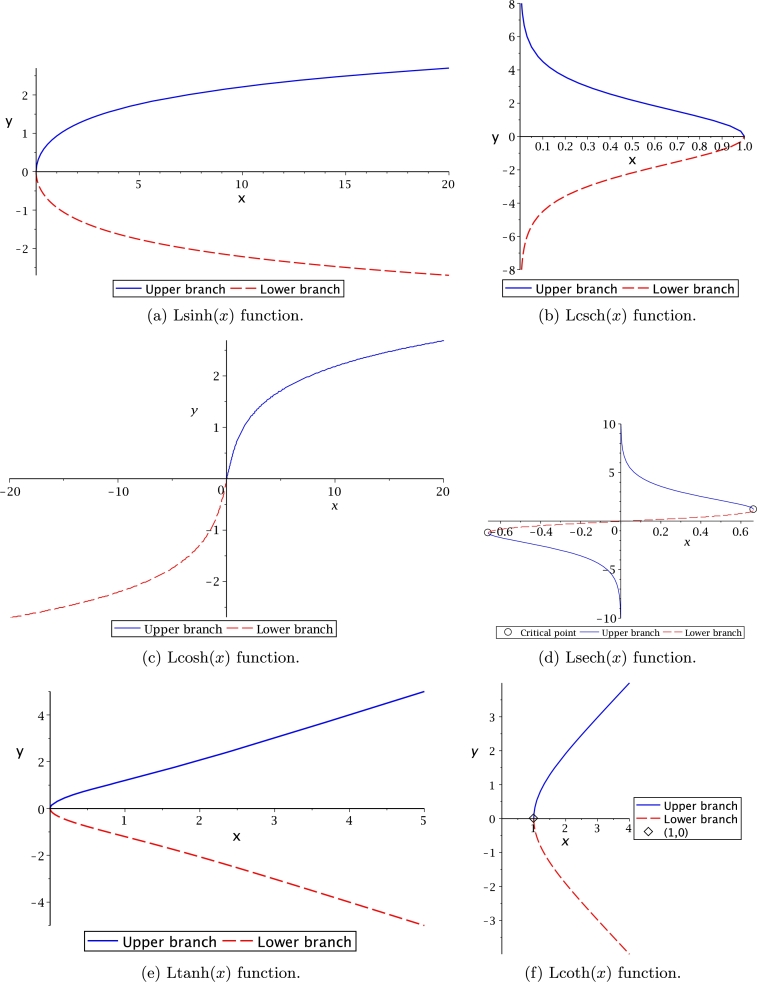
Hyperbolic Leal-functions.

It is important to remark that some of these functions are multivalued. Thus, notation (+) and (−) is used as superscript for the proposed transcendental function to denote the upper or lower branch, respectively. For the case of Lsech(*x*), the domain and range are bounded by limit values obtained using the critical points theory [62]. On the other hand, Lln(*x*) presents an asymptotic behaviour at the lower branch, obtained by the limits theory [62]. Ltan(*x*) also presented that behaviour. From this table, it is important to note that the proposed transcendental functions actually are the inverse function for very associated transcendental equations. The behaviour of Leal-functions derived from hyperbolic functions is shown in Fig. 3.

**Figure 4:**
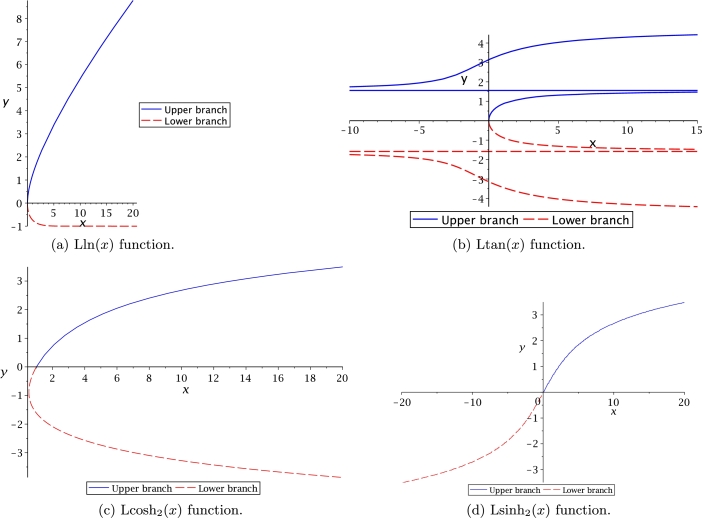
Leal-functions behaviour.

Fig. 4(a) displays Lln(*x*) as multivalued but not symmetrical, like W(x) function from Fig. 1(c) and Lsech(*x*) presented in Fig. 3(d). An interesting observation is that upper branch is monotonically increasing, while the lower branch behaves asymptotically having the limit y=−1. By comparison, Fig. 4(b) shows that Ltan(*x*) is symmetrical and asymptotic with limit y=±π/2 as principal branch. This asymptote is vertical at x=±nπ/2 for tan⁡(x) as depicted in Fig. 1(a); and horizontally for y=±nπ/2, n=[1,2,3,⋯], for tan⁡(y) as shown in Fig. 4(b). For both Leal-functions, these values were obtained with asymptotic techniques. At Fig. 4(c), it is shown that function domain for Lcosh${}_{2}(x)$ starts at (1,0), just like Lcoth(*x*). It is noteworthy to mention that all Leal-functions are described in the domain of real numbers, requiring further work to study the complex domain.

Table 3 presents the derivatives and integrals rules for Leal-functions. As occurs for elementary functions, superior derivatives and integrals can be obtained iteratively following the basic known rules of differential and integral calculus [62]. On one side, Leal-functions derivatives were obtained by implicit differentiation. On the other side, the integral rule for these functions can be obtained as follows:

(14) ∫y(x)dx=y(x)g(y(x))−∫g(y)dy,

where y(x) is the Leal-function to integrate and g(y) is the left hand side of the associated transcendental equation (see definitions of Leal-functions)

**Table 3:** Integration and differentiation rules for Leal-functions.

*y*(*x*)=	*y*′(*x*)=	∫*y*(*x*)*dx*=
Lsinh(*x*)	$\frac{1}{\text{sinh}(\text{Lsinh}(x))+\text{Lsinh}(x)\cosh(\text{Lsinh}(x))}$	$-\text{Lsinh}(x)\cosh(\text{Lsinh}(x))+\sinh(\text{Lsinh}(x))+\text{Lsinh}{\left(x\right)}^{2}\sinh(\text{Lsinh}(x))+C$
Lcosh(*x*)	$\frac{1}{\cosh(\text{Lcosh}(x))+\text{Lcosh}(x)\sinh(\text{Lcosh}(x))}$	$-\text{Lcosh}(x)\sinh(\text{Lcosh}(x))+\cosh(\text{Lcosh}(x))+\text{Lcosh}{\left(x\right)}^{2}\cosh(\text{Lcosh}(x))+C$
Ltanh(*x*)	$\frac{-1}{\tanh{\left(\text{Ltanh}(x)\right)}^{2}\text{Ltanh}(x)-\tanh(\text{Ltanh}(x))-\text{Ltanh}(x)}$	Ltanh(*x*)tanh2⁡(Ltanh(x))−(∑n=0∞22n(22n−1)B2n(Ltanh(x))2n+1(2n+1)!)+C
Lcsch(*x*)	$\frac{-1}{\text{csch}(\text{Lcsch}(x))(\coth(\text{Lcsch}(x))\text{Lcsch}(x)-1}$	Lcsch(*x*)csch2(Lcsch(x))−(∑n=0∞2(−1)2n−1(22n−1−1)B2n(Lcsch(x))2n+1(2n+1)!)+C
Lsech(*x*)	$\frac{-1}{\text{sech}(\text{Lsech}(x))(\text{Lsech}(x)\tanh(\text{Lsech}(x))-1)}$	Lsech(*x*)sech(Lsec(x)) 2−(∑n=0∞E2n(Lsech(x))2n+2(2n+2)(2n)!)+C
Lcoth(*x*)	$\frac{-1}{\coth{\left(\text{Lcoth}(x)\right)}^{2}\text{Lcoth}(x)-\coth(\text{Lcoth}(x))-\text{Lcoth}(x)}$	$\text{Lcoth}{\left(x\right)}^{2}\text{coth(Lcoth(}\text{x}\text{))}-(\frac{\sum_{n=0}^{\infty }2^{2n}{\left(-1\right)}^{2n}B_{2n}{\left(\text{Lcoth}(x)\right)}^{2n+1}}{(2n+1)!})+C$
Lln(*x*)	$\frac{\text{Lln}(x)+1}{\text{Lln}(x)\ln(\text{Lln}(x)+1)+\ln(\text{Lln}(x)+1)+\text{Lln}(x)}$	$\frac{1}{2}{\left(\text{Lln}(x)+1\right)}^{2}\text{Lln}(\text{Lln}(x)+1)+\frac{1}{4}\text{Lln}{\left(x\right)}^{2}-\frac{1}{2}\text{Lln}(x)-(\text{Lln}(x)+1)\ln(\text{Lln}(x)+1)+\ln(\text{Lln}(x)+1)-\frac{3}{4}+C$
Ltan(*x*)	$\frac{1}{\tan{\left(\text{Ltan}(x)\right)}^{2}\text{Ltan}(x)+\tan(\text{Ltan}(x))+\text{Ltan}(x)}$	$\text{Ltan}{\left(x\right)}^{2}\tan(\text{Ltan}(x))-(\frac{\sum_{n=0}^{\infty }2^{2n}(2^{2n}-1)|B_{2n}|{\left(\text{Ltan}(x)\right)}^{2n+1}}{(2n+1)!})+C$
Lsinh_2_(*x*)	$\frac{1}{1+\cosh({\text{Lsinh}}_{2}(x))}$	$\frac{1}{2}{\text{Lsinh}}_{2}{\left(x\right)}^{2}+{\text{Lsinh}}_{2}(x)\text{sinh}({\text{Lsinh}}_{2}(x))-\text{cosh}({\text{Lsinh}}_{2}(x))+C$
Lcosh_2_(*x*)	$\frac{1}{1+\sinh({\text{Lcosh}}_{2}(x))}$	$\frac{1}{2}{\text{Lcosh}}_{2}{\left(x\right)}^{2}+{\text{Lcosh}}_{2}(x)\text{cosh}({\text{Lcosh}}_{2}(x))-\sinh({\text{Lcosh}}_{2}(x))+C$

*E* are Euler numbers.

*B* are Bernoulli numbers.

The Euler number E(n) is defined by the exponential generating function [71]

(15) $$\frac{2}{\exp(x)+\exp(-x)}=\sum_{i=0}^{\infty }\frac{E_{i}t^{i}}{i!}.$$

Likewise, in [72], the Bernoulli numbers are defined by

(16) $$\sum_{0\leq i\leq k}\left(\begin{array}{c} k+1\\ i \end{array}\right)B_{i}=k+1.$$

A collection of PSEM approximations for Leal-functions are listed in Table 4 along with the power series expansions (PSE) for some of them at the initial region. This table shows (±) for symmetrical functions, upper, and lower branches. In the same way, asymmetrical transcendental functions Lsech(*x*) and Lln(*x*) exhibit different approximations for their upper and lower branches. PSE is a well established method from literature, then, we will focus on PSEM in the next section.

**Table 4:** PSEM and PSE approximations for Leal-functions. Plus sign means superior branch and minus sign means inferior branch.

∼Lsinh(*x*) = $\pm W(1.97955220294x^{\frac{9999}{10000}}-0.127018932445x^{\frac{3}{4}}+1.26880612005\ln(\frac{1}{2}x^{\frac{3}{10}}+1)),\;x>1.20$,
∼Lsinh(*x*) = $\pm (\sqrt{x}-\frac{1}{12}x^{3/2}+\frac{29x^{5/2}}{1440}-\frac{263x^{7/2}}{40320}+\frac{23479x^{9/2}}{9676800}+\cdots -\frac{481980717701293x^{19/2}}{12456458281864396800}),\;0\leq x\leq 1.20$,
∼Lcosh(*x*) = $W(1.935357823x^{\frac{99999}{100000}}+\frac{1}{2}x^{\frac{7}{10}}-0.394422973\ln(6.43591542x+1)),\;x>0.55$,
∼Lcosh(*x*) = $x-\frac{1}{2}x^{3}+\frac{17x^{5}}{24}-\frac{961x^{7}}{720}+\frac{116129x^{9}}{40320}+\cdots -\frac{1909820766285110401x^{19}}{6402373705728000},\;0\leq x\leq 0.55$,
∼Ltanh(*x*) = $\pm (\frac{853}{854}x+\frac{52}{45}\exp(-\frac{21}{13}x)(x^{\frac{7}{12}}-\frac{29}{73}\ln(\frac{3}{4}x+x^{2}+1)\exp(-x^{2}))),\;x>2$,
∼Ltanh(*x*) = $\pm (\sqrt{x}+\frac{1}{6}x^{3/2}+\frac{11x^{5/2}}{360}+\frac{17x^{7/2}}{5040}-\frac{281x^{9/2}}{604800}-\frac{44029x^{11/2}}{119750400}-\frac{12147139x^{13/2}}{130767436800}-\frac{2030761x^{15/2}}{784604620800}),\;0\leq x\leq 2$,
∼Lcsch(*x*) = $\pm \frac{275569}{13880}\exp(-\frac{42644}{18789}x^{\frac{9}{50}}-\frac{12362}{16313}x^{\frac{3}{2}}+\frac{3}{20}\ln(30x^{3}-\frac{131347}{3336}x^{6}+\frac{87}{10}x+1)-\frac{5899}{27158}\ln(40x^{\frac{5}{4}}+\frac{13}{10}x+1)),0\leq x\leq 1$,
∼Lsech^+^(*x*) = $-\frac{547}{465}\ln(1000000x-1)+\frac{83}{576}\ln(-\frac{257}{344}x+\frac{1}{2})-\frac{2563}{57}\ln(\frac{22}{2011}x+\frac{2}{3}),\;0\leq x\leq \alpha$,
∼Lsech^−^(*x*) = 0.234121102ln⁡(2.14673324x+1)−0.0879503835ln⁡(−1.50350706x+1)−0.34605317ln⁡(−1.15x+1),0≤x≤α,
∼Lcoth(*x*) = $\pm (\frac{968}{969}{\left(x^{2}-1\right)}^{\frac{1}{2}}+\ln({\left(x-1\right)}^{\frac{293}{2237}}{\left(x+1\right)}^{\frac{3069}{224}}{\left(x+2\right)}^{\frac{3162}{127}}{\left(x-\frac{1}{2}\right)}^{\frac{658}{1619}})-\ln({\left({\left(x-1\right)}^{\frac{1}{5}}+1\right)}^{\frac{939}{535}}{\left(x+\frac{3}{2}\right)}^{\frac{4128}{107}})),\;x\geq 1$,
∼Lln^+^(*x*) = $(-0.4563394683x^{0.9759932428}+\frac{3}{4}x^{\frac{99999}{100000}}){\left(W(\frac{3}{10}x^{0.5504791883})\right)}^{-1},\;x>2.00$,
∼Lln^+^(*x*) = $\sqrt{x}+x/4-\frac{x^{3/2}}{96}+\frac{59\;x^{5/2}}{92160}-\frac{x^{3}}{2880}+\frac{2783\;x^{7/2}}{20643840}-\frac{x^{4}}{24192}+\frac{1060117\;x^{9/2}}{118908518400}-\frac{x^{5}}{4838400},\;0\leq x\leq 2.00$,
∼Lln^−^(*x*) = $-\tanh(\frac{46003}{50177}\sqrt{x}+\frac{74539}{17155}\ln(\frac{67}{8000}x^{2}+1)),\;x>2.10$,
∼Lln^−^(*x*) = $-\sqrt{x}+x/4+\frac{x^{3/2}}{96}-\frac{59\;x^{5/2}}{92160}-\frac{x^{3}}{2880}-\frac{2783\;x^{7/2}}{20643840}-\frac{x^{4}}{24192}-\frac{1060117\;x^{9/2}}{118908518400}-\frac{x^{5}}{4838400},\;0\leq x\leq 2.10$,
∼Ltan(*x*) = $\pm \arctan\left(-\frac{13310221}{128764011}x^{\frac{19}{100}}+x^{\frac{6064391}{14247061}}+\frac{27964123x^{5/4}}{105243440}\right),\;x>1.20$,
∼Ltan(*x*) = $\pm (\sqrt{x}-\frac{1}{6}x^{3/2}+\frac{11x^{5/2}}{360}-\frac{17x^{7/2}}{5040}-\frac{281x^{9/2}}{604800}+\cdots -\frac{397228981889x^{19/2}}{121645100408832000}),\;0\leq x\leq 1.20$,
∼Lcosh_2_(*x*) = $-W(\cosh(x))+x-\frac{52055}{80137}\exp(-\frac{6079}{6700}x),\;x\geq 1$,
∼Lsinh_2_(*x*) = $-W(\sinh(x))+x+\frac{62020}{160123}x\exp(-\frac{13097}{10984}x),\;x\geq 0$.

## 5 PSEM solution procedure

This section presents the PSEM solution procedure for Lsinh (*x*), given that PSEM was employed for all the functions presented in this article, only the procedure to obtain Lsinh (*x*) is shown. The steps for the deduction of the other functions are presented at the end of this section in Table 5.

**Table 5:** PSEM procedure parameters.

Transcendental function	Coefficients of Taylor series	*x*_0_	Order	C.P. 1	C.P. 2	C.P. 3
Lsinh(*x*)	[0.9320200294,0.4098481265,−0.13539606952,0.067892662865,−0.0415700556,0.0286134875,⋯]	1	2	-	-	-
Lcosh(*x*)	[0.7650099546,0.5125074924,−0.180635222,0.07483331097,−0.02611498931,0.002427189707,⋯]	1	2	-	-	-
Ltanh(*x*)	[2.065338139,0.91159888000,0.0471728868,−0.0071551106,−0.0049239641,0.00298264714,⋯]	2	3	-	-	-
Lcsch(*x*)	[4.499913997,−12.85313302,59.07499089,−390.2580446,2901.393053,−23080.74415,⋯]	0.1	1	0.6	0.94	1
Lsec^+^(*x*)	[2.86155666,−4.77205201502,4.96659720520,−13.21674719,24.041733163,−71.4465220495,⋯]	0.325	2	0.6627	0.64	-
Lsech^−^(*x*)	[0.5248170331,1.52660640,1.94023078,5.485288521,17.1143005,60.0263269,⋯]	0.46	2	0.649	0.662743385	-
Lcoth(*x*)	[1.9150080482,+1.1485988053,−0.1374910206,0.0970398547,−0.0646876027,+0.0455669151,⋯]	2	4	1.01	1000	-
Lln^+^(*x*)	[2.432512797,0.514943443,−0.025684687,0.0041364036,−0.0008519024,0.0001976850,⋯]	3	2	-	-	-
Lln^−^(*x*)	[−0.956554813,−0.0397554809,0.017367901,−0.0048002818,0.0009815332,−0.0001888604,⋯]	3	1	-	-	-
Ltan(*x*)	[0.860333589,0.313970436,−0.145530879,0.0752504269,−0.0446189748,0.0298857708,⋯]	1	2	-	-	-
Lcosh_2_(*x*)	[0.7252637249,0.5584909162,−0.1110295782,0.03132750416,−0.012085069,0.00569946605,⋯]	2	0	1	-	-
Lsinh_2_(*x*)	[0.490073069,0.471140620,−0.02666426400,−0.00619994796,0.00168823363,0.0000538783,⋯]	1	1	-	-	-

$x_{0}$ is the Taylor series expansion point.

C.P. means cancellation point.

It is proposed to expand Lsinh(*x*) at $x_{0}=1$. Then, substituting $x=x_{0}$ into the associated transcendental equation (see Leal-function definitions) and solving, Lsinh(x)=±0.9320200294 is obtained. Now, using the positive solution and expanding in Taylor series [68], we obtain

(17) $$\begin{array}{ccc} \text{T(Lsinh}(x))\; & =\; & 0.932020029+0.4098481265(x-1)-0.13539607{\left(x-1\right)}^{2}\\ \; & \; & +0.067892663{\left(x-1\right)}^{3}-0.041570056{\left(x-1\right)}^{4}\\ \; & \; & +0.0286134875{\left(x-1\right)}^{5}+\cdots +\text{O}(x^{n}), \end{array}$$

where T(⋅) denotes the Taylor expansion. Now, we propose the following TF

(18) $$∼\text{Lsinh}(x)=W(a_{0}x^{\frac{9999}{10000}}+a_{1}x^{\frac{3}{4}}-a_{2}\ln(\frac{1}{2}x^{\frac{3}{10}}+1)),$$

where $a_{0}$, $a_{2}$, and $a_{3}$ are constants to be determined by PSEM.

Obtaining the Taylor series of (18) at x=1, yields

(19) $$\text{T(}∼\text{Lsinh}(x))=\frac{\beta }{{\left(1+W(\alpha )\right)}^{3}{\left(\alpha \right)}^{2}}+(\frac{\gamma }{(1+W{\left(\alpha \right)}^{3}){\left(\alpha \right)}^{2}})(x-1)+(\frac{\delta }{{\left(1+W(\alpha )\right)}^{3}{\left(\alpha \right)}^{2}}){\left(x-1\right)}^{2}+\cdots +\text{O}({\left(x-1\right)}^{n}),$$

where

$$\alpha =a_{0}+a1-0.405465108108a_{2},$$

$$\beta =(W(\alpha )+3(W{\left(\alpha \right)}^{2}+W{\left(\alpha \right)}^{3})+W{\left(\alpha \right)}^{4})a_{0}^{2}+(W(\alpha )+3(W{\left(\alpha \right)}^{2}+W{\left(\alpha \right)}^{3})+W{\left(\alpha \right)}^{4})a_{1}^{2}+(0.164401953893(W(\alpha )+W{\left(\alpha \right)}^{4})+0.493205861679(W{\left(\alpha \right)}^{2}+W{\left(\alpha \right)}^{3}))a_{2}^{2}+(2(W(\alpha )+W{\left(\alpha \right)}^{4})+6(W{\left(\alpha \right)}^{2}+W{\left(\alpha \right)}^{3}))a_{0}a_{1}-(0.810930216216(W(\alpha )+W{\left(\alpha \right)}^{4})+2.43279064865(W{\left(\alpha \right)}^{2}+W{\left(\alpha \right)}^{3}))a_{0}a_{2}-(0.810930216216(W(\alpha )+W{\left(\alpha \right)}^{4})+2.43279064865(W{\left(\alpha \right)}^{2}+W{\left(\alpha \right)}^{3}))a_{1}a_{2},$$

$$\gamma =(0.9999(W(\alpha )+W{\left(\alpha \right)}^{3})+1.9998W{\left(\alpha \right)}^{2})a_{0}^{2}+(0.75(W(\alpha )+W{\left(\alpha \right)}^{3})+1.5W{\left(\alpha \right)}^{2})a_{1}^{2}+(0.0405465108108(W(\alpha )+W{\left(\alpha \right)}^{3})+0.0810930216216W{\left(\alpha \right)}^{2})a_{2}^{2}+(1.7499(W(\alpha )+W{\left(\alpha \right)}^{3})+3.4998W{\left(\alpha \right)}^{2})a_{0}a_{1}-(0.505424561597(W(\alpha )+W{\left(\alpha \right)}^{3})+1.01084912319W{\left(\alpha \right)}^{2})a_{0}a_{2}-(0.404098831081(W(\alpha )+W{\left(\alpha \right)}^{3}+3.4998W{\left(\alpha \right)}^{2})a_{1}a_{2},$$

$$\delta =(-(4.9995\times 10^{-5}W(\alpha )+0.9999W{\left(\alpha \right)}^{2}+0.49995^{3}W{\left(\alpha \right)}^{3})a_{0}^{2}-(0.09375W(\alpha )+0.75W{\left(\alpha \right)}^{2}+0.375W{\left(\alpha \right)}^{3})a_{1}^{2}-(0.0162186W(\alpha )+0.04243721W{\left(\alpha \right)}^{2}+0.021218604W{\left(\alpha \right)}^{3})a_{2}^{2}-(0.093799995W(\alpha )+1.68744999W{\left(\alpha \right)}^{2}+0.843724995W{\left(\alpha \right)}^{3})a_{0}a_{1}+(0.0400202712W(\alpha )+0.28002054W{\left(\alpha \right)}^{2}+0.14001027W{\left(\alpha \right)}^{3})a_{0}a_{2}+(0.07801235W(\alpha )+0.30602470W{\left(\alpha \right)}^{2}+0.15301235W{\left(\alpha \right)}^{3})a_{1}a_{2}.$$

Equating the first three coefficients of (17) and (19), we obtain the following system of equations

(20) $$\begin{array}{cc} {\left(x-1\right)}^{0}:\; & \frac{\beta }{{\left(1+W(\alpha )\right)}^{3}{\left(\alpha \right)}^{2}}=0.932020029,\\ {\left(x-1\right)}^{1}:\; & \frac{\gamma }{{\left(1+W(\alpha )\right)}^{3}{\left(\alpha \right)}^{2}}=0.409848126480,\\ {\left(x-1\right)}^{2}:\; & \frac{\delta }{{\left(1+W(\alpha )\right)}^{3}{\left(\alpha \right)}^{2}}=-0.135396069523. \end{array}$$

**Figure 5:**
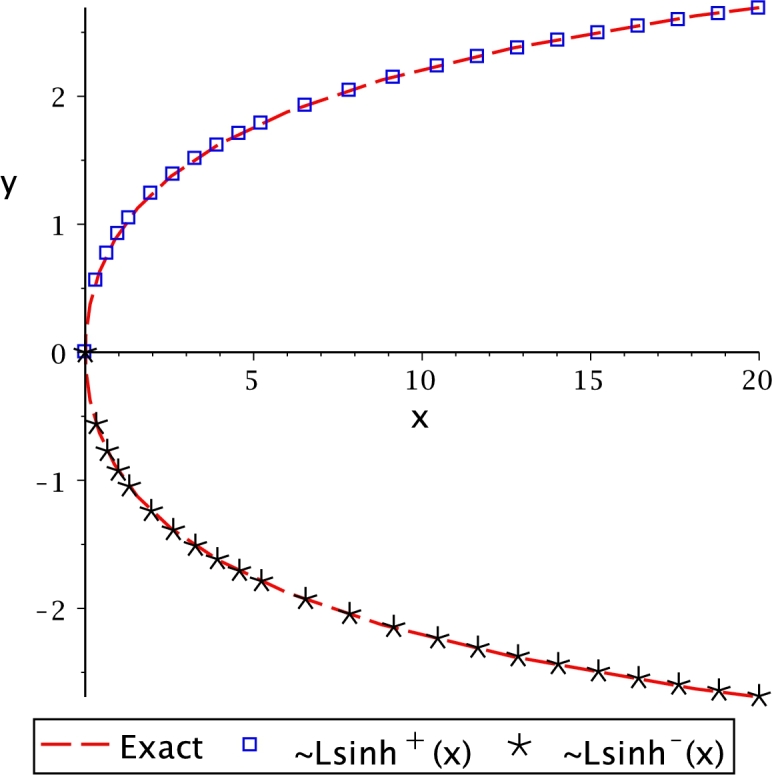
Comparison between ∼Lsinh(*x*) and exact value obtained using NR.

Solving the nonlinear system (20) and substituting $a_{0}$, $a_{1}$, and $a_{2}$ into (18), results

(21) $$∼\text{Lsinh}(x)=\pm W(1.97955220294x^{\frac{9999}{10000}}-0.127018932445x^{\frac{3}{4}}\;\;-1.26880612005\ln(\frac{1}{2}x^{\frac{3}{10}}+1)),$$

where (+) refers to the upper branch and (−) to the lower branch. Fig. 5 compares the approximation (21) against the numerical solution (obtained using Newton-Raphson), also known as the exact solution for practical purposes.

Table 5 resumes the PSEM procedure parameters to find the approximation for the rest of Leal-functions: Taylor series for each of these functions, employed expansion point, their expansion order, and employed cancellation points (CP) (if required). It is important to note that for determining the approximation of Lcsch(*x*), Lsech${}^{-}(x)$, Lcoth(*x*), and Lcosh${}_{2}(x)$ CP has been used to improve accuracy approximations. For instance, for Lcoth(*x*), seven adjustment constants were proposed. Then, to get a better precision to approximate Lcoth(*x*), two CP were employed (x=1.01 and x=1000). In this way, only a fourth-order Taylor expansion was necessary to obtain the rest of equations for the PSEM procedure.

**Figure 6:**
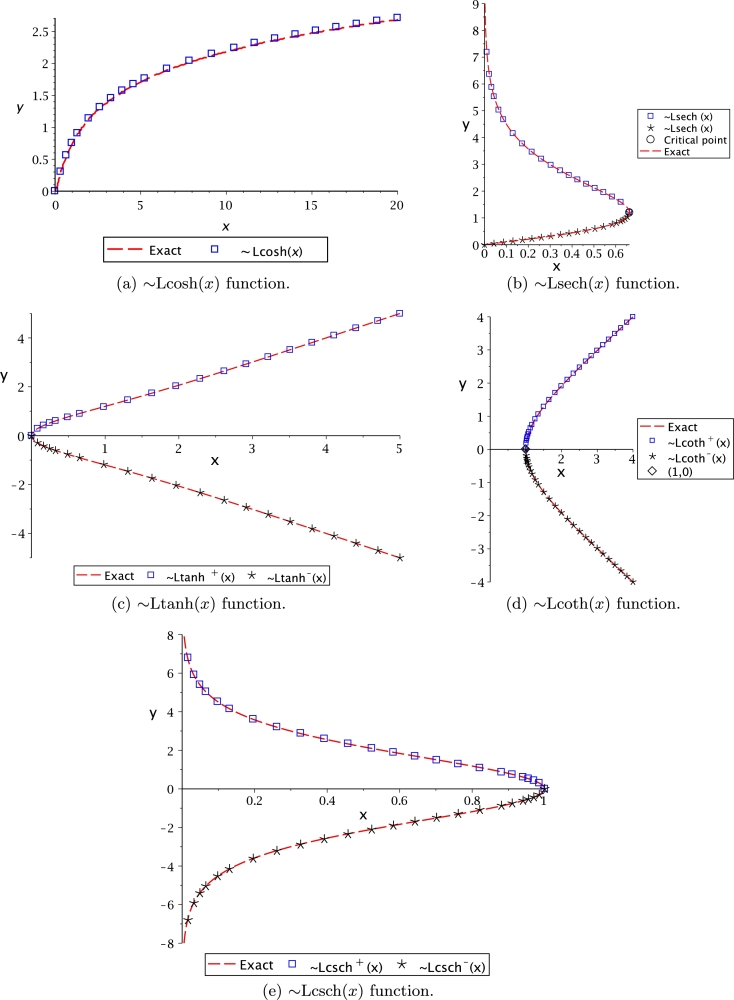
Hyperbolic related Leal-functions.

**Figure 7:**
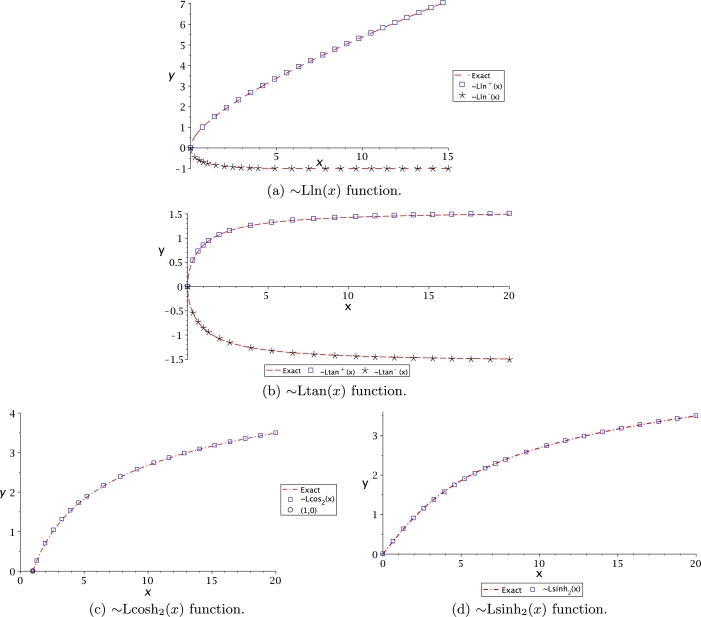
Other set of Leal-functions.

Figs. 6 and 7 show the approximations listed in Table 4, where the PSEM approximations are compared against the exact solution for the transcendental equation numerically obtained (NR). The approximation of Ltan(*x*) was only obtained for the main branch (multivalued).

## 6 Exploring the use of arguments in Leal-functions

The following procedure is applied to all the functions in this article. It is proposed to isolate *y* and obtain the graphic of both branches for:

(22) $$(-5y_{1}-4)\tan(-5y_{1}-4)=|\sin(\frac{3}{4})x|,$$

(23) $$(5y_{2}-4)\ln((5y_{2}-4)+1)=|\sin(\frac{3}{4})x|.$$

After a few algebraic steps, we can isolate *y* as follows

(24) $$y_{1}^{+}=\frac{{\text{Ltan}}^{+}(|\sin(\frac{3}{4})x|)+4}{-5},y_{1}^{-}=\frac{{\text{Ltan}}^{-}(|\sin(\frac{3}{4})x|)+4}{-5},$$

(25) $$y_{2}^{+}=\frac{{\text{Lln}}^{+}(|\sin(\frac{3}{4})x|)+4}{5},y_{2}^{-}=\frac{{\text{Lln}}^{-}(|\sin(\frac{3}{4})x|)+4}{5}.$$

**Figure 8:**
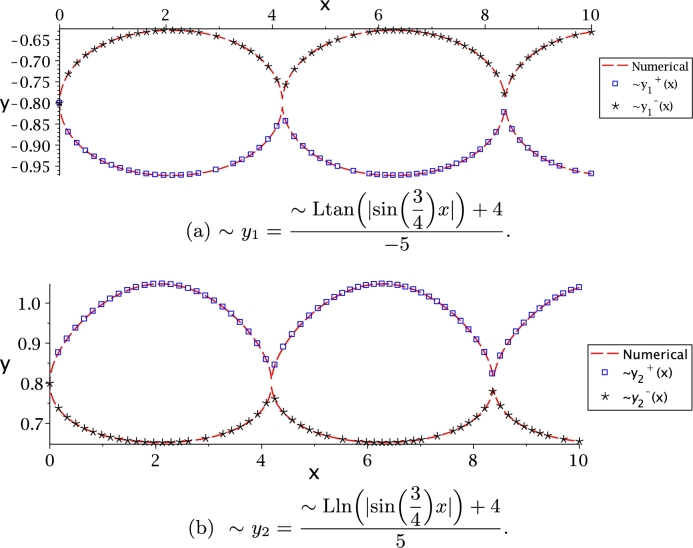
Comparison between numerical solution of *y*_1_, *y*_2_, and PSEM approximations.

The numerical solution of (22) and (23) matches perfectly the analytical solution (24) and (25), respectively (see Fig. 8). It is important to note how the trigonometric function introduces a periodicity that is observed in the exact solution, as well as in the respective approximation. Fig. 8(a) shows the symmetry for solution (24); this symmetry is due to the real branches of Ltan(*x*), while Fig. 8(b) shows that solution (25) is asymmetric due to Lln(*x*) asymmetry.

## 7 Applications to science and engineering

This section presents a set of case studies to exemplify how to use the new transcendental functions and show their wide potential application to science and engineering.

### 7.1 Case 1: applications to coastal structures

**Figure 9:**
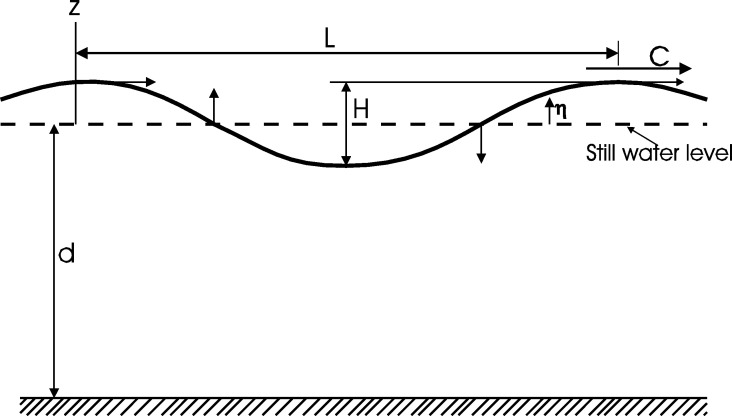
Definition of progressive surface wave parameters [18].

The fundamentals of wave mechanics and coastal processes at the practice of coastal engineering were introduced in [18]. Fig. 9 shows the definition for progressive surface wave parameters of a monochromatic wave travelling at a wave celerity (phase velocity), *C*; at a water depth, *d*; in a coordinate system *x*, *z*. *x* axis is the still water position; wave trough is at z=−d in meters [m]; z=η is the wave surface profile where *η* is a function of *x* and time *t*, in seconds [s]. *L* is the wave length and *H* the wave height, both given in [m].

Since the wave travels a distance *L* in [m] in one period *T* in [s], and celerity is given by

(26) $$C=\frac{L}{T},$$

where the wave length *L* is given as

(27) $$L=\frac{gT^{2}}{2\pi }\tanh\frac{2\pi d}{L},$$

and *g* is the gravity constant, in [m/s^2^].

The location of any water particle is (ζ,ε); *u* and *w* are the horizontal and vertical velocity components, respectively (see Fig. 9); *k* is the wave number at the horizontal and vertical components, given by

(28) $$k=\frac{2\pi }{L},$$

and *n* is the fraction of the mechanical energy in a wave that is transmitted forward each wave period given as

(29) $$n=\frac{1}{2}(1+\frac{2kd}{\text{sinh}(2kd)}).$$

In [18], the wave length, wave number, and the fraction of mechanical energy was calculated in a wave that is transmitted forward in each period for a wave in water a depth of 100 meters that has a period of 10 seconds and a height of 2 meters when it propagates in a 10 meter depth without refracting; assuming energy gains and losses can be ignored and a deep water wave has $L_{0}=156.1310$ m. The wavelength is obtained by (27). This case study uses Ltanh(*x*) function, solving (27) for *L*, yields:

(30) $$L=\frac{2\pi d}{{\text{Ltanh}}^{+}(\frac{4\pi^{2}d}{gT^{2}})}.$$

Considering T=10 s and d=10 m, we obtain Table 6, which is in good agreement with the phenomenon. Additionally, from Table 7, it can be noticed that relative error for *L*, *n*, and *k* are very low, in the order of 1E−3.

**Table 6:** Comparison between (30) using PSEM approximation and NR solution of (27).

Equation	*L* [m]	*n*(29)	*k* [m^−1^] (28)
(30)	92.5526	0.8740	0.0679
NR (27)	92.3738	0.8758	0.0673

**Table 7:** Relative errors.

*L* [m]	*n*(29)	*k* [m^−1^] (28)
1.93 × 10^−3^	2.06 × 10^−3^	8.92 × 10^−3^

### 7.2 Case 2: determination of mechanicals parameters of long transmission lines span with support at different levels

**Figure 10:**
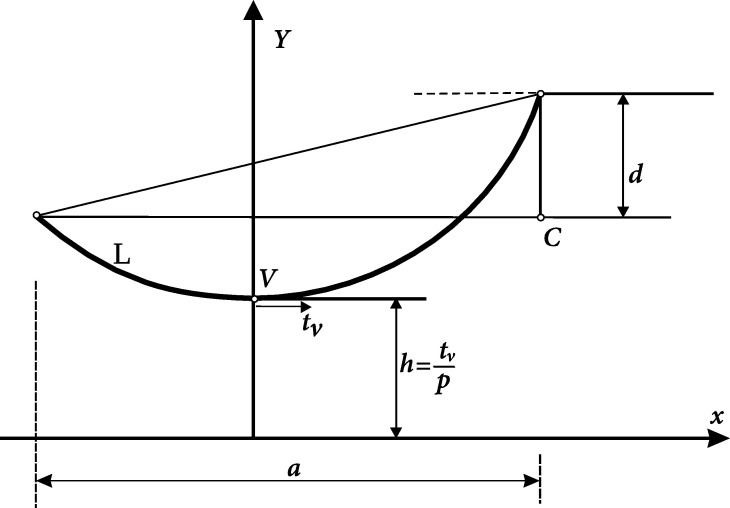
The conductor length in a transmission line span with support at different levels (inclined spans).

The tension at the vertex of a catenary curve in a transmission line $t_{v+15^{\circ }}$ and the total tension of the vertex $T_{v+15^{\circ }}$ are calculated in [15], both at 15 °C temperature. This problem supposed a cable of 800 meters long with support structures every 50 meters; such support points have different levels of altitude, as depicted in Fig. 10.

The conductor cable specifications are shown in Table 8.

**Table 8:** Technical information of wire conductor.

Specifications		Values
Composition		
	Aluminium	54× 3.376 mm
	Steel	7× 3.376 mm
Cross sections		
	Aluminium	483.42 mm^2^
	Steel	62.64 mm^2^
	Total	546.06 mm^2^
Copper equivalent cross section		304.03 mm^2^
Steel core diameter		10.135 mm^2^
Cable diameter		30.378 mm^2^
Weight		
	Aluminium	1.826 kg/km
	Steel	488 kg/km
Break Strength		15.536 kg
Elastic modulus		6.860 kg/mm^2^
Thermal expansion coefficient by temperature degree		19.35 × 10^−6^
Electrical resistance at 20 °C		0.0597 Ω/km

This case study calculates the tension at the vertex of a cable shaped as a catenary, with the same power transmission line $t_{v+15\;{}^{\circ}\text{C}}$ and total tension of the vertex $T_{v+15\;{}^{\circ}\text{C}}$, both at 15 °C as mentioned earlier. In addition, we take into consideration that the conductor is located, at least, 500 meters of altitude above the sea level. With the specifications mentioned in Table 8, the unitary weight of the cable *ω* = 0.003344 kg/m/mm^2^ in [15] was determined employing the approximations presented in this article.

Fig. 10 presents the schematics of the power transmission line, depicting the involved variables for the catenary and tension of the vertex calculations. The expression that relates cable longitude *L* at temperature $L_{+15^{\circ }}=806.79$ m, the support structure unleveled points *d*, and power line longitude *a* is

(31) $$\frac{\sqrt{L^{2}-d^{2}}}{a}=\frac{\sinh(z)}{z},$$

with

(32) $$z=\frac{a}{2h},h=\frac{t_{v}}{\omega },T_{v}=t_{v}S,$$

where *h* is the distance from vertex to origin, in [m]; $t_{v}$ is the tension at *V*, in [kg/m/mm^2^]; *ω* is the conductor weight, in [kg/m/mm^2^]; $T_{v}$ is the total tension of the catenary vertex in [kg]; and *S* is the transversal section of the conductor, in [mm^2^].

Using the function Lcsch(*x*), and solving for *z* in (31), we obtain

(33) $$z={\text{Lcsch}}^{+}(\frac{a}{\sqrt{L^{2}-d^{2}}}),\;\;d<L.$$

Substituting the calculated values ω=0.003344 kg/m/mm^2^, S=546.06 mm^2^, as reported in [15], into (33) and proposing the numerical values for the same regulation temperature, *a* = 700 m, *d* = 85 m, *L* = 708.69 m, the value of *z* is determined. Resulting values are displayed in Table 9 (see (32)).

**Table 9:** Comparison between solution (33) using approximation ∼Lcsch^+^(*x*) and NR solution of (31).

Equations	*z*	*h* [m]	*t*_*v*_ [kg/mm^2^]	*T*_*v*_ [kg] (32)
(33)	0.2713	1290.0848	4.3140	2355.7266
NR (31)	0.2714	1289.6094	4.3126	2354.8586

The values calculated from *z* are listed in Table 9. Also, is included a comparison between *z* values obtained with (33) against the NR numerical solution of (31). The absolute relative error for *z*, *h*, $t_{v}$, and $T_{v}$ using ∼Lcsch^+^(*x*) with (33) and (32) is small as shown in Table 10.

**Table 10:** Absolute relative error obtained using PSEM approximation.

Equation	*z*	*h* [m]	*t*_*v*_ [kg/mm^2^]	*T*_*v*_ [kg] (32)
(33)	3.69 × 10^−4^	3.69 × 10^−4^	3.25 × 10^−4^	3.69 × 10^−4^

### 7.3 Case 3: the planar one-dimensional Bratu equation

Gelfand equation (Bratu's equations in one dimension) is very important in the fields of science and engineering, such as, the Chandrasekar model for the universe expansion [73], chemical reactor theory, and nanotechnology [74], [75], [76], among others. Bratu's equation is given by

(34) $$u^{\prime \prime }+\lambda \exp(u)=0,\;0<x<1,$$

with boundary conditions

(35) u(0)=u(1)=0.

Given the solution for (34) is important, several authors have proposed approximate solutions employing different analytical and semi-analytical methodologies [77], [78], [79], among others. The planar one-dimensional Bratu problem is given by (34) with exact solution [80], [81] given by:

(36) $$u(x)=2\ln(\frac{\cosh(\alpha )}{\cosh(\alpha (1-2x))}),$$

where *α* satisfies the transcendental equation

(37) $$\cosh(\alpha )=\frac{4}{\sqrt{2\lambda }}\alpha .$$

**Figure 11:**
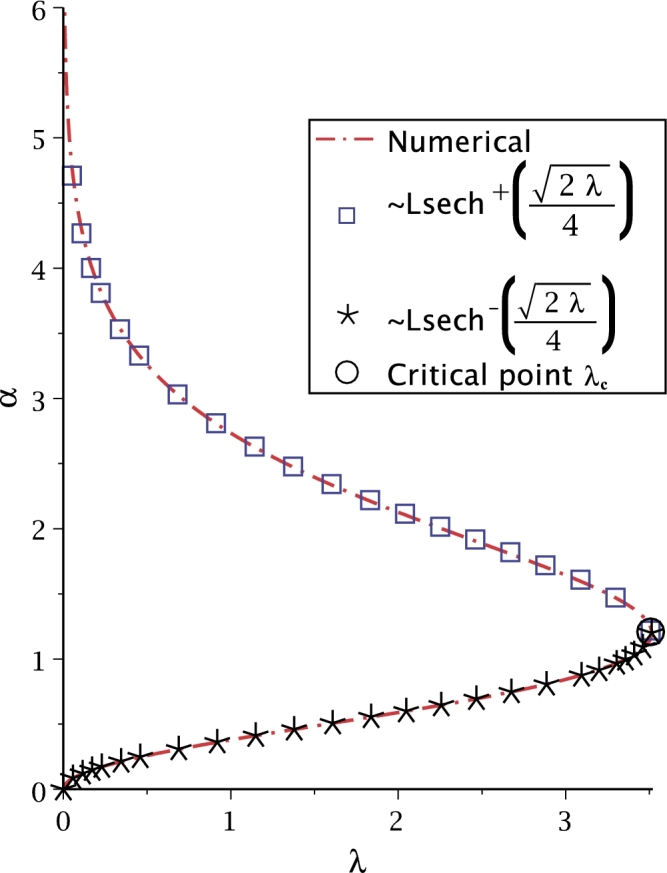
Comparison between (37) and (38).

The numerical solution of (37) was obtained using the modified Newton-Raphson method, recently in [82]. In [83], the critical point for (37) was calculated resulting $\lambda_{c}=3.513830719$ (see Fig. 11). At this value there is only one solution. There are two possible solutions for λ<3.513830719, and no solution for λ>3.513830719. In this work, equation (37) is solved employing Lsech(*x*) function and using the procedure depicted in the first case study. Thus, the solution is expressed as

(38) $$\alpha =\text{Lsech}(\frac{\sqrt{2\lambda }}{4}).$$

Fig. 11 shows the numerical solution (using NR) of the transcendental equation (37) (dash-dot), related to the solution of Bratu's equation, which depends on *α* and *λ*, besides of being multivalued. Furthermore, the critical point of u(x) when λ=3.513830719 is $u(\frac{1}{2})=1.227$ [84] (empty circle). Additionally, the comparison of (37) against the solution presented here (38) for upper and lower branches (square box and asterisks, respectively) is depicted in Fig. 11.

Thus, substituting (38) into (36), the exact solution of Bratu's equation can be rewritten as

(39) $$u(x)=2\ln(\frac{\cosh(\text{Lsech}(\frac{\sqrt{2\lambda }}{4}))}{\cosh(\text{Lsech}(\frac{\sqrt{2\lambda }}{4})(1-2x))}),$$

avoiding the application of NR.

**Figure 12:**
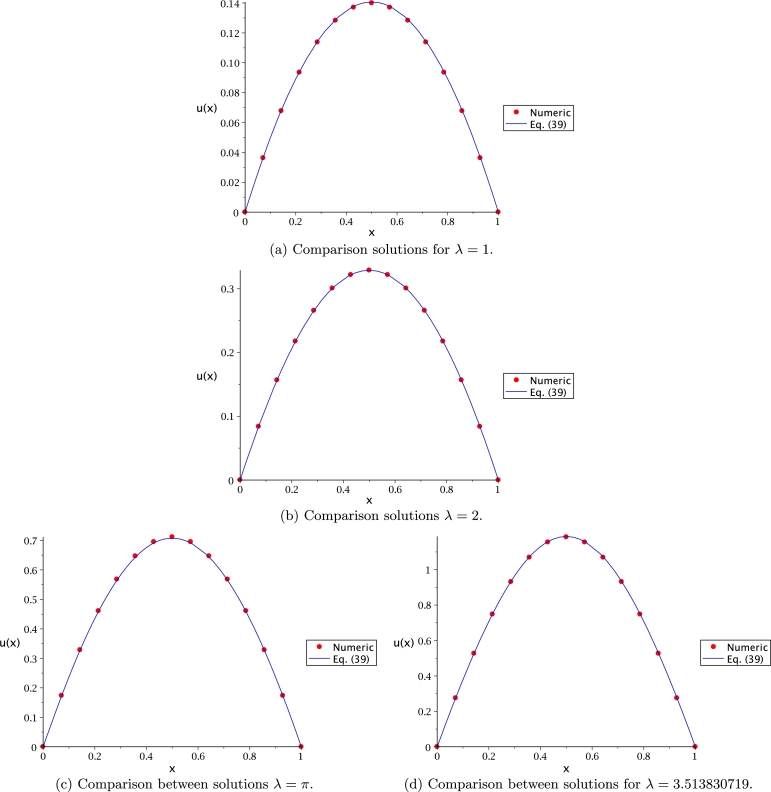
Comparison between the exact (39) lower solution for *λ* = [1,2,*π*,3.513830719], using ∼Lsech(*x*).

Fig. 12 depicts the solution obtained by (39) (using ∼Lsech(*x*)) compared to the numerical solution for λ=[1,2,π,3.513830719] (using NR). This figure shows that, for the different values of *λ* using ∼Lsech(*x*), remains close to the numerical solution for all *x* interval. It is important to remark that the numerical solutions were obtained using a trapezoid method that use Richardson extrapolation enhancement [85] as a built-in command in Maple.

**Figure 13:**
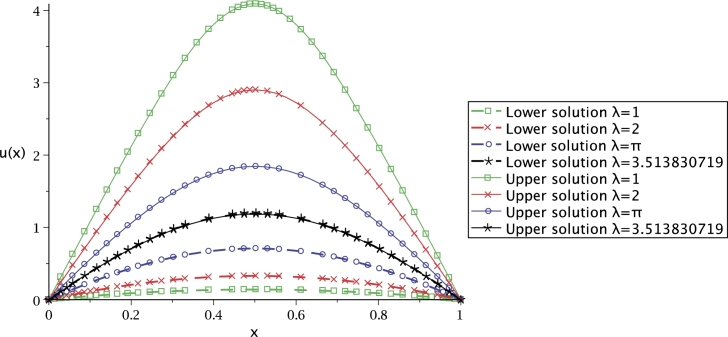
Upper and lower solution for the 1-D Bratu equation with *λ* = [1,2,*π*,3.513830719].

The solution plot of Bratu's equation employing (39) (using ∼Lsech(*x*)) is presented in Fig. 13, showing the upper and lower solutions for λ=[1,2,π,3.513830719]. From this figure, it can be seen that, as *λ* increases, the solution branches approach until they overlap at λ=3.513830719; being the critical point where solution is univalued.

## 8 A proposal for the practical implementation of Leal-functions

The computational implementation of the Leal-functions requires capability to increase the number of significant digits for the approximations reported in Table 4, as required for the specific implementation. Therefore, we propose to use the approximations from Table 4 as a source for the initial point for the Halley's method [86], [87]. The Halley's method is a recurrence formula to approximate equations and its formulation is given by:

(40) $$x_{k+1}=x_{k}-\frac{f(x_{k})}{f^{\prime }(x_{k})-\frac{1}{2}\frac{f(x_{k})f^{\prime \prime }(x_{k})}{f^{\prime }(x_{k})}},\;k=0,1,2,\cdots ,$$

where f(x) is the equation to solve.

**Figure 14:**
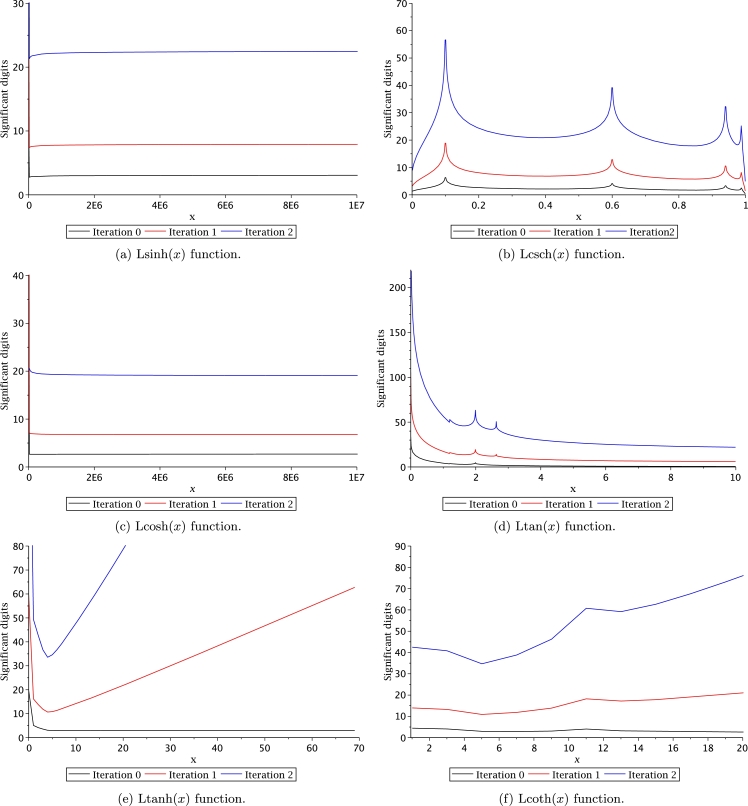
Significant digits for Halley's method iterations.

**Figure 15:**
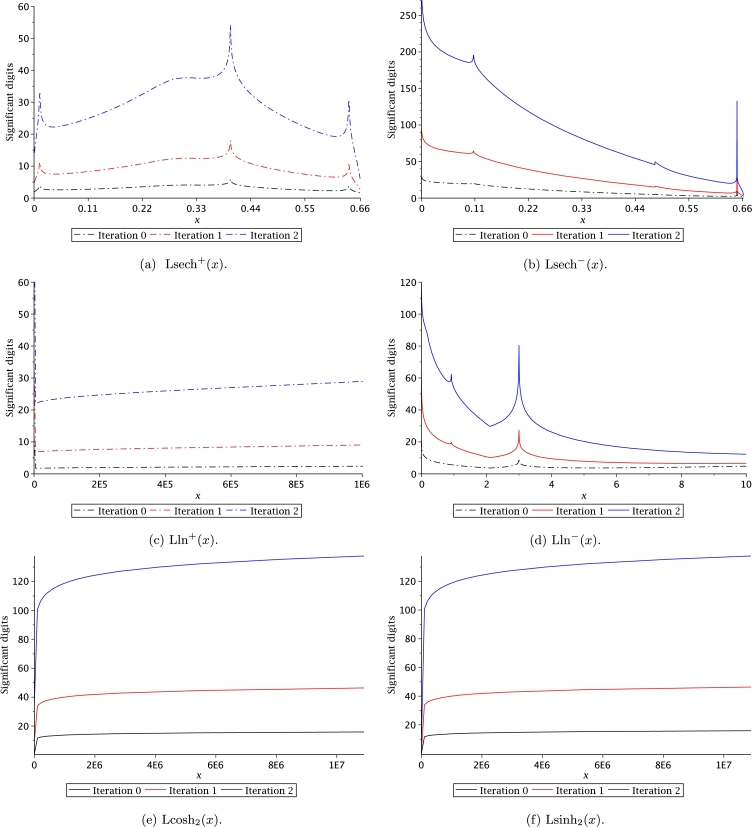
Significant digits for Halley's method iterations.

In order to solve transcendental equations based on the definition of Leal-functions (see Section 4), we will employ the approximations of Table 4 to provide the initial value $x_{0}$ for Halley's method. Figs. 14 and 15 present the significant digits (SD) (as reported by [64]) for two iterations of Halley's method. It is important to remark that iteration zero means that we use directly the approximations of Table 4 to provide an initial approximation. As expected, we can establish from these figures that iteration zero exhibits a relatively poor accuracy; nonetheless, after two iterations of Halley's method the number of significant digits reach a compatibility with the double float (64-bit IEEE 754) format of C/C++, which provides 15 SD. In fact, for some approximations after two iterations, the number of SD reach more than 40 digits. Therefore, users can increase the number of SD [64] for the approximations in Table 4 by increasing the number of Halley's iterations.

## 9 Computation time and discussion

In this work, we proposed new transcendental functions with their respective derivatives and integrals, which are listed in Table 3. Given that the Leal family of functions is new, there are no numerical/analytical methods that would allow to evaluate them efficiently. For this reason, a series of approximations were proposed employing PSEM. One of the advantages when using PSEM is that this method allows obtaining handy accurate approximations, simple to compute, with a wide domain of convergence. Therefore, PSEM approximations allow performing fast and efficient numerical evaluations that will decrease computing times. It is important to remark that the PSEM approximations for Leal-functions depicted in Table 4 are constituted by combining known transcendental functions and polynomials aiming to simplify coding new functions in any computer language. If users require more accuracy a combination with Helley's method can be used; increasing the number of significant digits. This means that users can increase the Leal-function evaluations accuracy to fulfil specific application requirements.

**Figure 16:**
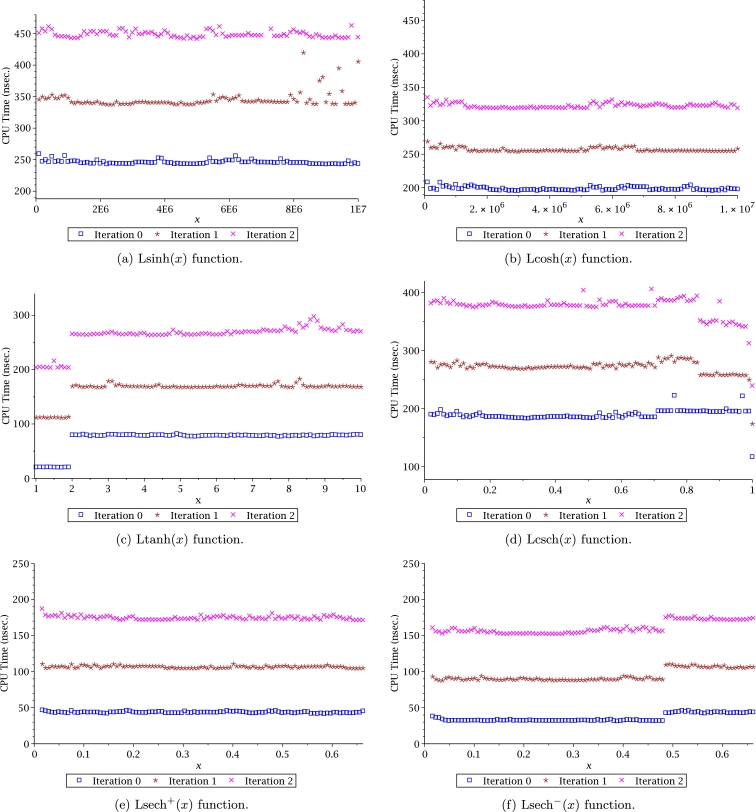
Computation time for Halley's method to evaluate Leal-functions.

**Figure 17:**
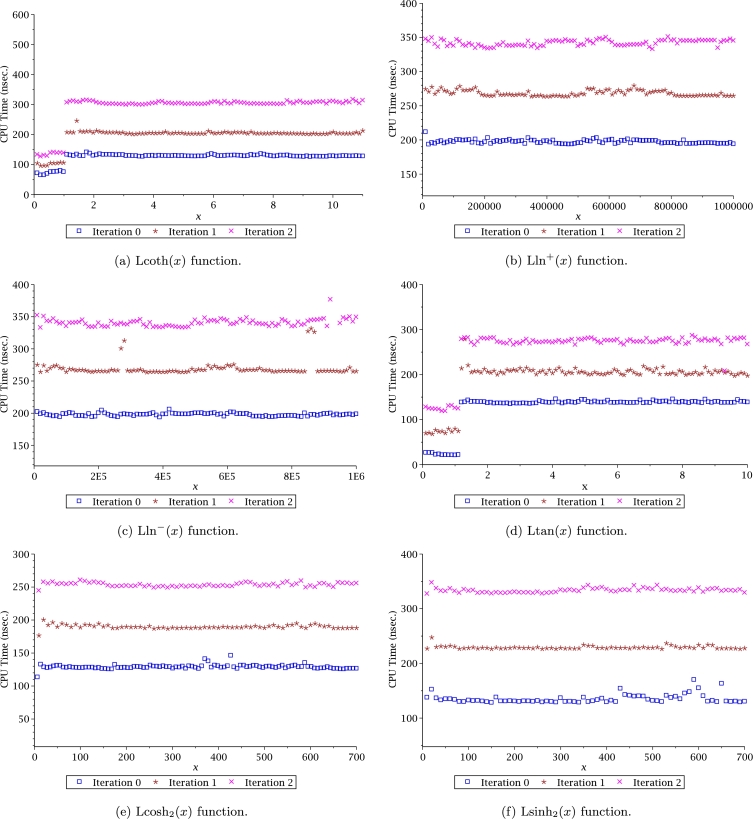
Computation time for Halley's method to evaluate Leal-functions.

Computation time (CT) is an important issue of any algorithm to evaluate a function. For the case of Leal-functions, it is also important to measure the approximations CT in combination with the Halley's method. CT was measured with Fortran 77/90, using as compiler the gcc 5.4.0 with level 3 of optimization. The computer used for the simulations was an Intel Core Pentium(R) CPU 2117Ux2 running at 1.80 GHz under Linux Ubuntu 16.04. In order to circumvent the operative system instabilities, the time measurement was determined by the average time of 10 million evaluations for each point. Results are depicted in Figs. 16 and 17, where the CT was measured for iterations 0, 1 and 2. We can conclude from such figures that computation time for iteration 2 requires between 150 nsec to 450 nsec, which is still practical for large scale simulations considering that for iteration 2, the number of SD are between 20 to 40 digits (see Figs. 14 and 15).

In the first case study, the application of Ltanh(*x*) for a coastal engineering problem was presented, where it was necessary to solve for *L* variable which determines the wave length of the deep weather wave. In this problem was demonstrated that, employing Ltanh${}^{+}(x)$, it is possible to solve analytically the equation of the deep weather wave and obtain highly accurate results using its PSEM approximation.

The second case study, demonstrated that it is possible to employ Lcsch(*x*) function to analytically solve a mechanical-electrical engineering problem since it was possible to solve the equation that relates the variable *z* (geometry related), cable longitude *L*, the structure support points *d*, and the overhead longitude *a* of the power lines (see (33)). Additionally, employing ∼Lcsch${}^{+}(x)$ it is possible to obtain highly accurate results. Nevertheless, just as depicted in the first and second study case, if more accuracy is required, it is recommended to use the PSEM approximated result as starting point for Halley's method.

For the third case study, Bratu's equations were solved using Lsech(*x*). For comparison purposes, the numerical solution (considered as the exact solution) was obtained by Runge-Kutta employing Maple 15 dsolve command; with a tolerance error of $1\times 10^{-12}$ for the inferior solution with λ=[1,2]. For λ>π it was necessary to set the error tolerance to $10^{-2}$ due to the numerical difficulty that this particular problem represents. Remarking that the analytical solution proposed here does not exhibit the mentioned numerical issue. Furthermore, Fig. 13 shows the behaviour of upper and lower solution for λ=[1,2,π,3.513830719] using (39). As *λ* increases, the solution branches tend to approach, one in front of the other. At critical point λ=3.513830719, the two solutions overlap. In this graphic, a comparison for the upper solution against the numerical solution was not possible because Maple 15 inter-constructed algorithm is limited to calculate only a single solution (the inferior branch). In order to circumvent such issue, the exact value from the lower branch was obtained solving (37) employing NR and using this result to evaluate (36).

It is important to highlight that the Leal family of function can be extended to solve other transcendental equations. Therefore, in Table 11 it is introduced another set of new transcendental functions along with their respective derivatives and integrals. Finally, it is worthy to remark that Leal-functions users could propose their own functions and perform their approximations using the methodology presented in this work.

**Table 11:** Complementary set of Leal-Functions.

Transcendental function	Transcendental equation g(y)=x	*y*′(*x*)	∫*y*(*x*)*dx*
*y*(*x*) = Lsin(*x*)	y(x)sin⁡(y(x))=x	$\frac{1}{\sin(\text{Lsin}(x))+\text{Lsin}(x)\cos(\text{Lsin}(x))}$	$-\sin(\text{Lsin}(x))+\text{Lsin}(x)\cos(\text{Lsin}(x))+\text{Lsin}{\left(x\right)}^{2}\sin(\text{Lsin}(x))+C$
*y*(*x*) = Lcos(*x*)	y(x)cos⁡(y(x))=x	$\frac{1}{\cos(\text{Lcos}(x))-\text{Lcos}(x)\sin(\text{Lcos}(x))}$	$-\cos(\text{Lcos}(x))-\text{Lcos}(x)\sin(\text{Lcos}(x))+\text{Lcos}{\left(x\right)}^{2}\cos(\text{Lcos}(x))+C$
*y*(*x*) = Lsec(*x*)	y(x)sec⁡(y(x))=x	$\frac{1}{\sec(\text{Lsec}(x))(\text{Lsec}(x)\tan(\text{ Lsec}(x))+1)}$	$\text{Lsec}{\left(x\right)}^{2}\text{sec(Lsec(}\text{x}\text{)) }-(\frac{\sum_{n=0}^{\infty }{\left(-1\right)}^{n}E_{2n}{\left(\text{Lsec}(x)\right)}^{2n+2}}{(2n+2)(2n)!})+C$
*y*(*x*) = Lcsc(*x*)	y(x)csc⁡(y(x))=x	$\frac{-1}{\csc(\text{Lcsc}(x))(\text{ Lcsc}(x)\cot(\text{Lcsc}(x))-1)}$	$\text{Lcsc}{\left(x\right)}^{2}\text{csch}(\text{Lcsc}(x))-(\frac{\sum_{n=0}^{\infty }2{\left(-1\right)}^{n+1}(2^{2n-1}-1)B_{2n}{\left(\text{Lcsc}(x)\right)}^{2n+1}}{(2n+1)!})+C$
*y*(*x*) = Lcot(*x*)	y(x)cot⁡(y(x))=x	$\frac{-1}{\cot{\left(\text{Lcot}(x)\right)}^{2}\text{Lcot }(x)-\cot(\text{Lcot}(x))+\text{Lcot}(x)}$	$\text{Lcot}{\left(x\right)}^{2}\text{coth(Lcot(}\text{x}\text{))}-(\frac{\sum_{n=0}^{\infty }2^{2n}{\left(-1\right)}^{n}B_{2n}{\left(\text{Lcot}(x)\right)}^{2n+1}}{(2n+1)!})+C$
*y*(*x*) = Ltanh_2_(*x*)	y(x)+tanh⁡(y(x))=x	$\frac{-1}{\tanh{\left({\text{Ltanh}}_{2}(x)\right)}^{2}-2}$	$\frac{1}{2}{\text{Ltanh}}_{2}{\left(x\right)}^{2}+{\text{Ltanh}}_{2}(x)\tanh({\text{Ltanh}}_{2}(x))-\ln(\text{cosh}({\text{Ltanh}}_{2}(x)))+C$
*y*(*x*) = Lcsch_2_(*x*)	*y*(*x*)+ csch(*y*(*x*))=*x*	$\frac{-1}{\text{ csch(Lcsch}{}_{2}(x)\text{)coth(Lcsch}{}_{2}(x)\text{)}-1}$	$\frac{1}{2}{\text{Lcsch}}_{2}{\left(x\right)}^{2}+{\text{Lcsch}}_{2}(x)\text{csch}({\text{Lcsch}}_{2}(x))-\ln(\frac{\text{cosh}({\text{Lcsch}}_{2}(x)-1)}{\text{sinh}({\text{Lcsch}}_{2}(x))})+C$
*y*(*x*) = Lsech_2_(*x*)	*y*(*x*)+sech(*y*(*x*))=*x*	$\frac{1}{\text{sech(Lsech}{}_{2}(x))\text{tanh(Lsech}{}_{2}(x)\text{) }-1}$	$\frac{1}{2}{\text{Lsech}}_{2}{\left(x\right)}^{2}+{\text{Lsech}}_{2}(x)\text{sech}({\text{Lsech}}_{2}(x))-\arctan(\text{arcsinh}({\text{Lsech}}_{2}(x)))+C$
*y*(*x*) = Lcoth_2_(*x*)	y(x)+coth⁡(y(x))=x	$\frac{-1}{\text{ coth(Lcoth}{{}_{2}(x))}^{2}-2}$	$\frac{1}{2}{\text{Lcoth}}_{2}{\left(x\right)}^{2}+{\text{Lcoth}}_{2}(x)\coth({\text{Lcoth}}_{2}(x))-\ln({\text{Lcoth}}_{2}(x))+C$
*y*(*x*) = Ltan_2_(*x*)	y(x)+tan⁡(y(x))=x	$\frac{1}{\tan{\left({\text{Ltan}}_{2}(x)\right)}^{2}+2}$	$\frac{1}{2}{\text{Ltan}}_{2}{\left(x\right)}^{2}+{\text{Ltan}}_{2}(x)\tan({\text{Ltan}}_{2}(x))+\ln(\text{cos}({\text{Ltan}}_{2}(x)))+C$
*y*(*x*) = Lsin_2_(*x*)	y(x)+sinh⁡(y(x))=x	$\frac{1}{1+\cos({\text{Lsin}}_{2}(x))}$	$\frac{1}{2}{\text{Lsin}}_{2}{\left(x\right)}^{2}+\cos({\text{Lsin}}_{2}(x))+{\text{Lsin}}_{2}(x)\sin({\text{Lsin}}_{2}(x))+C$
*y*(*x*) = Lcos_2_(*x*)	y(x)+cos⁡(y(x))=x	$\frac{-1}{\sin({\text{Lcos}}_{2}(x)-1)}$	$\frac{1}{2}{\text{Lcos}}_{2}{\left(x\right)}^{2}-\sin({\text{Lcos}}_{2}(x))+{\text{Lcos}}_{2}(x)\cos({\text{Lcos}}_{2}(x))+C$
*y*(*x*) = Lsec_2_(*x*)	y(x)+sec⁡(y(x))=x	$\frac{1}{1+\sec({\text{Lsec}}_{2}(x))\tan({\text{Lsec}}_{2}(x))}$	$\frac{1}{2}{\text{Lcsc}}_{2}{\left(x\right)}^{2}+\frac{{\text{Lcsc}}_{2}(x)}{\sin({\text{Lcsc}}_{2}(x))}-\ln(\csc({\text{Lcsc}}_{2}(x))-\cot({\text{Lcsc}}_{2}(x)))+C$
*y*(*x*) = Lcsc_2_(*x*)	y(x)+csc⁡(y(x))=x	$\frac{-1}{\csc({\text{Lcsc}}_{2}(x))\cot({\text{Lcsc}}_{2}(x))-1}$	$\frac{1}{2}{\text{Lcsc}}_{2}{\left(x\right)}^{2}+\frac{{\text{Lcsc}}_{2}(x)}{\sin({\text{Lcsc}}_{2}(x))}-\ln(\csc({\text{Lcsc}}_{2}(x))-\cot({\text{Lcsc}}_{2}(x)))+C$
*y*(*x*) = Lcot_2_(*x*)	y(x)+cot⁡(y(x))=x	$\frac{-1}{\cot{\left({\text{Lcot}}_{2}(x)\right)}^{2}}$	$\frac{1}{2}{\text{Lcot}}_{2}{\left(x\right)}^{2}-({\text{Lcot}}_{2}(x))\cot({\text{Lcot}}_{2}(x))-\ln(\sin({\text{Lcot}}_{2}(x)))+C$

A potential application for one of the functions listed in Table 11 is for the case of Lcsc(*x*) which can be found in the field of acoustics. Its application is in the analysis of frequency and phase response due to sound waves pressure for different incidence angles over the tube microphone capsules. In [88], the frequency and phase response curves are described by

(41) $$\frac{P(\theta )}{P_{0}}=\frac{\sin(\frac{\pi L}{\lambda }(1-\cos(\theta ))}{\frac{\pi L}{\lambda }(1-\cos(\theta ))},$$

where

• P(θ) is the microphone output at a given angle of sound incidence,
• $P_{0}$ is the microphone output along principal axis at zero degree of incidence,
• *λ* is the wavelength,
• *L* is the tube length,
• *T* is the incidence sound angle.

Therefore, it is possible to solve for any variable found at the *sine* function argument of (41). For instance, using Lcsc(*x*) to solve for *θ* in (41), yields:

(42) $$\theta =\arccos(\frac{\pi L-\lambda \text{Lcsc}(\frac{P_{0}}{P(\theta )})}{\pi L}).$$

Note that for the approximating Leal-functions associated to trigonometric functions is a complex challenge given the fact that such functions are multivalued and exhibit an undetermined number of real branches. Our research was focused in a deliberated manner to work in the domain of real numbers with the end to bound the research; nevertheless, we believe that it is necessary to perform an in-depth study in the analysis of Leal-functions to characterize and gain a better knowledge of their behaviour in the domain of complex numbers. The approximations presented in Table 4 describe by symmetries the complete behaviour of the proposed Leal-functions. Nonetheless, Lcosh${}_{2}(x)$ requires future work to approximate its lower branch.

Further work is required to improve the analytical approximations obtained in this work for the transcendental functions by modifying the PSEM procedure parameters or using other approximative methods [89]. On the other side, it is important to develop numerical techniques to allow the digital/hardware (built-in inside float point units (FPU)) implementation of Leal-functions in order to allow development of new and interesting software applications. We can conclude from the case studies, and from the algebraic properties of Leal-functions, that the field of application for our proposal will have an interesting impact in all areas of physics and mathematics providing new tools to scientist and engineers researching for new mathematical models and novel analytical/numerical analysis to be applied in the simulation, design, and implementation of new theories and technological innovations. Further research is required to explore the potential application of Leal-functions to the variational principle, artificial neural networks, among other areas.

Table 12 provides a set of new analytical variable isolations examples that were considered impossible, until now. Although, further future research is required to investigate the family of Leal-functions, and its properties, to find different ways to perform variable isolations. For instance, if we want to isolate y(x) from y(x)arccosh(y(x))=Q(x), using relation 2 from Table 12, we obtain y(x) = cosh(Lcosh(Q(x))), Q(x)>0.

**Table 12:** Some variable isolation with Leal-functions.

Relation	Solution for	Solution	Restriction
1	*αy*(*x*)f(*β*(*y*(*x*)))=*Q*(*x*)	$y(x)=\frac{1}{\beta }\text{Lf}(\frac{\beta Q(x)}{\alpha })$	$\frac{\beta }{\alpha }>0$
2	*y*(*x*)f^−1^(*y*(*x*))=*Q*(*x*)	*y*(*x*)=*f*(Lf((*Q*(*x*))))	*Q*(*x*)>0
3	$\alpha y(x)\ln({\left(1+y(x)\right)}^{\beta })=Q(x)$	$y(x)=\text{Lln}(\frac{Q(x)}{\alpha \beta })$	$\frac{Q(x)}{\alpha \beta }>0$
4	*α* + *y*(*x*)*f*(*y*)=*Q*(*x*)	*y* = *Lf*(*Q*(*x*)−*α*)	*Q*(*x*)−*α* > 0
5	y(x)(sin⁡(αy(x))sin⁡(βy(x))+cosh⁡(βy(x)cosh⁡(βy(x)))=Q(x)	$y(x)=\frac{1}{\alpha -\beta }\text{Lcos}((\alpha -\beta )Q(x))$	*α* − *β* > 0
6	y(x)exp⁡(y(x))−y(x)=Q(x)	y=ln⁡(1+Lln(Q(x)))	*Q*(*x*)>0
7	y(x)sin⁡(y(x))cos⁡(y(x))=Q(x)	$y(x)=\frac{1}{2}\text{Lsin}(4Q(x))$	*Q*(*x*)>0
8	−y(x)ln⁡(1−y(x))=Q(x) *Q*(*x*)<0	*y* = −Lln(−*Q*(*x*))	*Q*(*x*)<0
9	y(x)sin⁡(−y(x))=Q(x)	*y*(*x*)=Lsin(−*Q*(*x*))	*Q*(*x*)>0

• *f* is any trigonometric or hyperbolic function.

• $f^{-1}$ represent the inverse (arc) of a trigonometric or hyperbolic function.

• Lf(*x*) represents any Leal-function.

• *α* and *β* are constants.

Finally, Table 13 presents a set of identities that relates some Leal-functions employing known trigonometry identities and hyperbolic functions. It is interesting to observe, from the expressions listed in this table, that there is a connection, theoretically, between these functions. This implies future work to further understand, in a more in-depth way, such functions and their relationships.

**Table 13:** New identities.

Lsin(*x*) = *i*Lsinh(−*x*)	Lcsc(*x*) = *i*Lcsch(*x*)
Lcos(*x*) = *i*Lcosh(−*ix*)	Lsec(*x*) = *i*Lsech(−*ix*)
Ltan(*x*) = *i*Ltanh(−*x*)	Lcot(*x*) = *i*Lcoth(*x*)

## 10 Concluding remarks

This work proposed a new family of transcendental functions denominated Leal, which exhibit algebraic properties that allow to perform new analytical variable isolations, where only was possible to know the variable value employing numerical methods. Additionally, a set of analytical approximations containing known polynomial and transcendental functions was proposed, allowing to evaluate the Leal-functions. Moreover, we provided the differentiation and integration rules for the Leal-functions; where, the superior order derivatives and integrals can be obtained using conventional calculus rules. In civil and electrical engineering, the catenary is a phenomenon under study in the design of bridges structures and transmission power lines, among others. In coastal engineering, the use of transcendental functions has a remarkable importance in the study of sea waves, which permits the design of constructions and structures of sea ports, among others. For this reason, we think that there are several possible applications to discover where Ltanh(*x*) and Lcsch(*x*) can be applied for analysis in other areas of knowledge. Lsech(*x*) function was employed to solve and important Physics problem, the planar one-dimensional Bratu Equation. Additionally, it was demonstrated the use of Lsech(*x*) to solve, analytically, the two branches or solution curves of the mentioned problem. From the three case studies, it is concluded that Leal-functions may be employed to solve different nonlinear algebraic problems and nonlinear differential equations. Furthermore, it is important to note that the proposed Leal-functions can be extended to solve different transcendental equations; aiming to increase the set of Leal-functions and make them available for their application in different areas of science and engineering. Finally, it will be interesting to include this new functions in commercial or open source mathematical software like: Maple, Mathematica, Matlab, GNU Octave, among others.

## Declarations

### Author contribution statement

H. Vazquez-Leal: Conceived and designed the experiments; Performed the experiments; Analyzed and interpreted the data; Contributed reagents, materials, analysis tools or data; Wrote the paper.

M.A. Sandoval-Hernandez: Conceived and designed the experiments; Performed the experiments; Analyzed and interpreted the data; Contributed reagents, materials, analysis tools or data.

U. Filobello-Nino: Performed the experiments; Analyzed and interpreted the data; Contributed reagents, materials, analysis tools or data.

### Funding statement

Dr Sandoval-Hernandez was supported by Secretary of Public Education of Mexico (10.13039/100010096SEP) through the grant 2703 E476300.0275652.

### Declaration of interests statement

The authors declare no conflict of interest.

### Additional information

No additional information is available for this paper.
